# Measurement Properties of Smartphone Approaches to Assess Diet, Alcohol Use, and Tobacco Use: Systematic Review

**DOI:** 10.2196/27337

**Published:** 2022-02-17

**Authors:** Louise Thornton, Bridie Osman, Katrina Champion, Olivia Green, Annie B Wescott, Lauren A Gardner, Courtney Stewart, Rachel Visontay, Jesse Whife, Belinda Parmenter, Louise Birrell, Zachary Bryant, Cath Chapman, David Lubans, Tim Slade, John Torous, Maree Teesson, Pepijn Van de Ven

**Affiliations:** 1 The Matilda Centre for Research in Mental Health and Substance Use The University of Sydney Sydney Australia; 2 School of Medicine and Public Health The University of Newcastle Newcastle Australia; 3 School of Public Health and Community Medicine University of New South Wales Kensington Australia; 4 Galter Health Sciences Library & Learning Center Northwestern University Feinberg School of Medicine Chicago, IL United States; 5 National Drug Research Institute Curtin University Perth Australia; 6 School of Health Sciences The University of New South Wales Sydney Australia; 7 Priority Research Centre for Physical Activity and Nutrition The University of Newcastle Newcastle Australia; 8 Beth Israel Deaconness Medical Centre Harvard Medical School Boston, MA United States; 9 Health Research Institute University of Limerick Limerick Ireland

**Keywords:** smartphone, app, alcohol, smoking, diet, measurement, mobile phone

## Abstract

**Background:**

Poor diet, alcohol use, and tobacco smoking have been identified as strong determinants of chronic diseases, such as cardiovascular disease, diabetes, and cancer. Smartphones have the potential to provide a real-time, pervasive, unobtrusive, and cost-effective way to measure these health behaviors and deliver instant feedback to users. Despite this, the validity of using smartphones to measure these behaviors is largely unknown.

**Objective:**

The aim of our review is to identify existing smartphone-based approaches to measure these health behaviors and critically appraise the quality of their measurement properties.

**Methods:**

We conducted a systematic search of the Ovid MEDLINE, Embase (Elsevier), Cochrane Library (Wiley), PsycINFO (EBSCOhost), CINAHL (EBSCOHost), Web of Science (Clarivate), SPORTDiscus (EBSCOhost), and IEEE Xplore Digital Library databases in March 2020. Articles that were written in English; reported measuring diet, alcohol use, or tobacco use via a smartphone; and reported on at least one measurement property (eg, validity, reliability, and responsiveness) were eligible. The methodological quality of the included studies was assessed using the Consensus-Based Standards for the Selection of Health Measurement Instruments Risk of Bias checklist. Outcomes were summarized in a narrative synthesis. This systematic review was registered with PROSPERO, identifier CRD42019122242.

**Results:**

Of 12,261 records, 72 studies describing the measurement properties of smartphone-based approaches to measure diet (48/72, 67%), alcohol use (16/72, 22%), and tobacco use (8/72, 11%) were identified and included in this review. Across the health behaviors, 18 different measurement techniques were used in smartphones. The measurement properties most commonly examined were construct validity, measurement error, and criterion validity. The results varied by behavior and measurement approach, and the methodological quality of the studies varied widely. Most studies investigating the measurement of diet and alcohol received *very good* or *adequate* methodological quality ratings, that is, 73% (35/48) and 69% (11/16), respectively, whereas only 13% (1/8) investigating the measurement of tobacco use received a *very good* or *adequate* rating.

**Conclusions:**

This review is the first to provide evidence regarding the different types of smartphone-based approaches currently used to measure key behavioral risk factors for chronic diseases (diet, alcohol use, and tobacco use) and the quality of their measurement properties. A total of 19 measurement techniques were identified, most of which assessed dietary behaviors (48/72, 67%). Some evidence exists to support the reliability and validity of using smartphones to assess these behaviors; however, the results varied by behavior and measurement approach. The methodological quality of the included studies also varied. Overall, more high-quality studies validating smartphone-based approaches against criterion measures are needed. Further research investigating the use of smartphones to assess alcohol and tobacco use and objective measurement approaches is also needed.

**International Registered Report Identifier (IRRID):**

RR2-https://doi.org/10.1186/s13643-020-01375-w

## Introduction

### Background

Traditional measurement techniques to assess health behaviors can be difficult and burdensome for individuals, clinicians, and researchers alike and are often subject to problems such as recall bias and forgotten information [1]. Novel measurement techniques are needed to increase compliance and accuracy with recording data, reduce respondent burden, and increase the quality and detail of health behavior information. Smartphones may present an opportunity to do just this.

Smartphones have become an integral part of the lives of many people [2], and users often use their smartphones and smartphone apps to record and measure a range of health behaviors [3]. In addition, the standard features of smartphones (ie, sensors, such as accelerometers, gyroscopes, and light sensors) allow these devices to continuously monitor contexts of users (eg, activity, location, and environment). Data from these sensors can be collected *passively*, without the active involvement of the user, and generate information about some behaviors with little burden [4]. Unfortunately, the ability to accurately measure key health behaviors using smartphones is currently hampered by a lack of understanding of the validity and reliability of the approaches used.

Consumption behaviors, such as dietary intake, alcohol use, and tobacco smoking, are typically measured using approaches prone to bias. For instance, diet is often assessed using food diaries that require participants to record everything they eat and drink for a period. This approach requires participants to be literate and highly motivated, and research has shown that the quality of food records declines considerably over time [5]. Retrospective recall methods are also commonly used for these behaviors. These methods often require multiple administrations to accurately capture variations in behavior over time [5,6], rely heavily on the memory of participants and interviewer training, and may be affected by social desirability bias, particularly for smoking and alcohol use. In addition, the accuracy of these self-report approaches is dependent on the ability of participants to accurately estimate portion sizes (or standard drinks) and, as such, often suffer from underreporting of behaviors [5,7,8].

Furthermore, traditional methods to objectively measure consumption behaviors are often burdensome and costly to administer. Weighed food records, for example, where food to be consumed and any waste left over are weighed and recorded, have been shown to be a valid method of recording dietary intake. However, outside of a laboratory setting, this approach is extremely burdensome and impractical [5]. In addition, although the *gold standard* doubly labeled water method (where isotopes in water provided to participants are used for tracing purposes) can accurately estimate the energy intake of participants, the approach requires multiple urine, saliva, or blood samples to be taken; is costly; requires sophisticated equipment; and is valid only among weight-stable participants. Therefore, it is only feasible within specialized research laboratories and not for use in clinical settings or by consumers themselves [5]. Although devices to objectively measure alcohol and tobacco use via expired breath ethanol and expired carbon monoxide (CO) are readily available for purchase, they must be regularly and properly calibrated to produce accurate results. Furthermore, as these behaviors often occur outside of the home and in social situations, their use may not be practical or acceptable in free-living conditions.

Given the ubiquitous and portable nature of smartphones, their powerful computing abilities, built-in cameras and sensors, and the social acceptance of their use in almost all situations, accurate smartphone measurement could offer solutions to many of the issues associated with traditional approaches to measure diet, alcohol, and tobacco use. Although several reviews of both published literature and mobile apps available in the marketplace have examined the efficacy of apps to help improve diet, alcohol use, and tobacco use, only 1 review to date has specifically focused on the measurement properties of smartphone-based approaches to measure any of these behaviors [9]. As such, there is a limited understanding of how these 3 behaviors might be validly and reliably measured using smartphones [10-13].

### Objectives

This study aims to systematically review the existing literature on the measurement properties of smartphone-based approaches to assess diet, alcohol use, and tobacco use. The specific objectives of this review are as follows:

1. To identify and describe the ways in which diet, alcohol use, and tobacco use have been measured using smartphones
2. To describe and critically evaluate the available evidence on the measurement properties of these approaches
3. To provide recommendations on the most suitable and effective ways of measuring diet, alcohol use, and tobacco use with smartphones

## Methods

### Overview

This review was conducted in accordance with the published review protocol [14] and the PRISMA (Preferred Reporting Items for Systematic Review and Meta-Analyses) guidelines [15]. It is part of a larger systematic review that examines the measurement properties of smartphone approaches to assess 6 key health behaviors (physical activity, sedentary behavior, sleep, diet, alcohol use, and tobacco use). Owing to the large number of eligible studies identified in this larger review, only those studies that examined consumption behaviors (ie, diet, alcohol use, and tobacco use) were included in the current review to allow for adequate description and discussion of the approaches identified and their associated measurement properties.

### Search Strategy and Selection Criteria

A research librarian (ABW) searched Ovid MEDLINE, Embase (Elsevier), Cochrane Library (Wiley), PsycINFO (EBSCOhost), CINAHL (EBSCOHost), Web of Science (Clarivate), SPORTDiscus (EBSCOhost), and IEEE Xplore Digital Library for research describing the measurement properties of smartphone-based approaches to assess at least one of the 6 key health behaviors. All databases were searched on March 1, 2020. A date limit was applied from 2007 to present, as 2007 is the year in which the first *smartphones* (ie, mobile phones with large capacitive touchscreens using direct finger input, as opposed to a stylus or keypad) were released. An example search strategy developed for MEDLINE is shown in Multimedia Appendix 1. Published studies with any type of study design, involving participants of all ages, were eligible for inclusion. Included articles were required to be in English language, peer-reviewed studies of human participants, describe a smartphone-based approach to assess diet, alcohol use, and tobacco use and to report on at least one measurement property of this approach identified in the Consensus-Based Standards for the Selection of Health Measurement Instruments (COSMIN) Taxonomy of Measurement Properties (Table 1).

**Table 1:** Consensus-Based Standards for the Selection of Health Measurement Instruments taxonomy of measurement properties^a^.

Domain	Domain description	Measurement properties
Reliability	Degree to which the measurement is free from measurement error	Internal consistency Reliability Measurement error
Validity	Degree to which an outcome measure measures the construct it purports to measure	Content validity (including face validity) Construct validity (including structural validity, hypotheses testing, and cross-cultural validity) Criterion validity
Responsiveness	Ability of an outcome measure to detect change over time	Responsiveness

^a^See Consensus-Based Standards for the Selection of Health Measurement Instruments definitions of domains, measurement properties, and aspects of measurement properties [16] for full descriptions and definitions of measurement properties.

Studies were excluded if they described the feasibility of the measurement approach only, described the measurement properties of using text messaging only to measure behaviors, or described the measurement properties of a wearable device (eg, Fitbit [Fitbit Inc]) alone.

### Data Extraction and Screening

All identified studies were exported into Endnote (version 8) to remove duplicates. Records were then uploaded to the Covidence Systematic Review software (Veritas Health Innovation) for screening. Authors participating in the screening, full-text review, and data extraction process participated in training sessions where multiple reviewers independently reviewed and discussed a selection of papers to ensure consistency across reviewers. Titles and abstracts were first screened by 1 reviewer (RV, JW, CS, LT, BO, LB, LG, OG, BP, or JT). Records were excluded if it was clear from the title and abstract that they did not examine the measurement properties of a smartphone-based approach to measure diet, tobacco, or alcohol. A total of 8 members of the research team (OG, CS, JW, LT, BO, ZB, KC, and RV) then participated in full-text screening of results, with the full text of potentially relevant studies independently assessed for eligibility by 2 members of this group, and any disagreements were resolved with the assistance of a third researcher. LT, BO, CS, or OG extracted data using a standardized form. Further details of the data extraction are included in the published protocol [14].

### Data Analysis

The primary outcomes of interest were the measurement properties of smartphone-based approaches to assess diet, alcohol use, and tobacco use. Specifically, we investigated, as reported, the internal consistency, reliability, measurement error, content validity, construct validity (including convergent validity), criterion validity, and responsiveness of the approaches identified. As there is currently no agreed-upon gold standard method for self-reported measurement of diet, alcohol use, or tobacco use, only studies in which the smartphone-based approach was compared with an objective measure of the behavior (eg, weighed food records and observed number of drinks or cigarettes consumed) were classified as investigating criterion validity. The smartphone-based approach was compared with a self-report measure, even if it was described as a gold standard method by the authors, the paper was classified as an investigating construct, specifically convergent validity.

A narrative synthesis of the included studies was undertaken for diet, alcohol use, and tobacco use separately, grouped according to the type of measurement approach used, which included *self-report approaches*, where participants were asked to actively enter self-report information about their behaviors; *active objective approaches*, where participants were asked to actively provide an objective measure of their behavior (eg, taking a photo of their food); and *passive objective approaches*, where data generated by smartphone sensors were collected without the active involvement of the participant and used to generate information about behaviors. The methodological quality of the included studies was assessed using the COSMIN Risk of Bias checklist [17]. The COSMIN Risk of Bias checklist was designed to assess the methodological quality of studies investigating the measurement properties of patient-reported outcome measures. It specifies several standards for design requirements and preferred statistical methods when assessing different measurement properties. The methodological quality of each study was evaluated by rating all standards for each measurement property investigated on a 4-point Likert scale. A standard can be rated as *very good* (there is evidence that the standard is met or when a preferred method was optimally used), *adequate* (it can be assumed that the standard is met or when the preferred method was used, but it was not optimally applied) *doubtful* (it is unclear whether the standard is met or unclear if a preferred method was used), or *inadequate* (there is evidence that the standard is not met or when the preferred method was not used). The overall quality of a study is determined by taking the lowest rating of any standard [17].

## Results

### Overview

**Figure 1:**
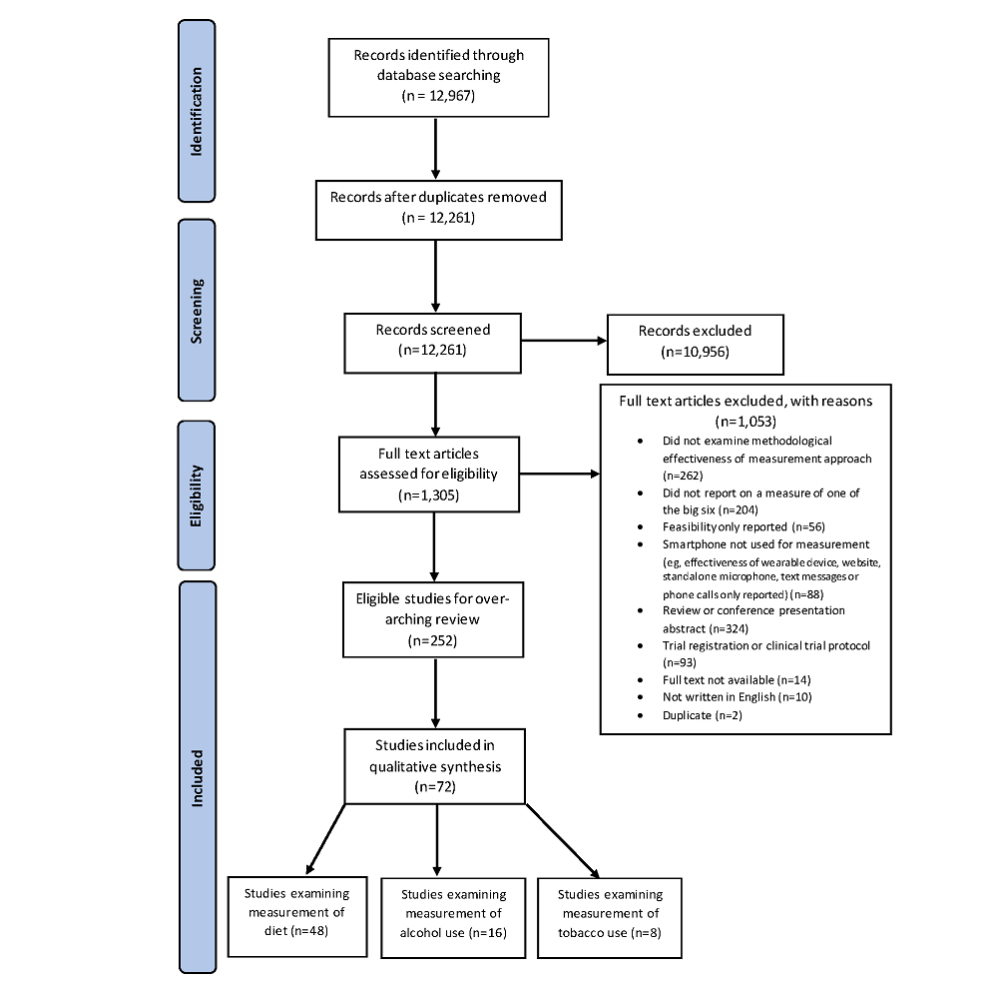
PRISMA (Preferred Reporting Items for Systematic Reviews and Meta-Analyses) flowchart.

Of 12,967 identified records, 1305 (10.06%) were independently fully reviewed by 2 reviewers. Agreement between reviewers was 83.22% (1086/1305). A total of 72 studies were ultimately included in the current review. These 72 studies involved 4732 participants and were most commonly conducted in the United States (27/72, 38%), European countries (9/72, 13%), the United Kingdom (6/72, 8%), and Australia (13/72, 18%). As shown in Figure 1, up to 67% (48/72) papers examined the measurement of diet, 22% (16/72) examined alcohol use measurement, and 11% (8/72) examined measurement of tobacco use. The details of the identified smartphone-based measurement approaches are discussed below.

### Diet

#### Overview

Overall, 67% (48/72) of the papers examined the measurement properties of a smartphone-based approach to assess diet (n range 0-203; 63.77% of participants in included studies were female; age range of participants 3-75 years). The key characteristics of these studies are detailed in Table 2 (for full study details, see Multimedia Appendix 2 [3,18-85]). Of the studies, 58% (28/48) described self-report approaches, whereas 42% (20/48) investigated active objective approaches. No studies have identified that used passive objective approaches to measure diet. The most commonly assessed measurement properties were construct validity, measurement error, and criterion validity.

**Table 2:** Key characteristics of studies examining the measurement of diet via a smartphone.

Study	Country	App name	Publicly available	Measurement approach	Measurement properties assessed					
					Reliability	Measurement error	Construct validity	Criterion validity	Responsiveness	Risk of bias
Ahmed et al [40]	Canada	MyFitnessPal	Yes	Self-report	—^a^	✓	—	✓	—	Very good
Ali et al [27]	United Kingdom	NR^b^	NR	Self-report	—	✓	✓	—	—	Doubtful
Ambrosini et al [26]	Australia	Easy diet diary	Yes	Self-report	—	✓	✓	—	—	Very good
Ashman et al [57]	Australia	DietBytes	NR	Active objective	—	✓	✓	—	—	Very good
Béjar [25]	Spain	e-EPIDEMIOLOGY	NR	Self-report	—	—	✓	—	—	Very good
Béjar [25]	Spain	e-12HR^c^	NR	Self-report	—	—	✓	—	—	Very good
Béjar et al [39]	Spain	e-12HR	NR	Self-report	—	—	✓	—	—	Very good
Béjar et al [36]	Spain	e-12HR	NR	Self-report	—	—	✓	—	—	Very good
Boushey et al [55]	United States	N/A^d^	N/A	Active objective	—	✓	—	✓	—	Adequate
Bruening et al [42]	United States	devilSPARC^e^	NR	Self-report	—	—	✓	—	—	Inadequate
Bucher et al [23]	Switzerland	e-CA^f^	NR	Self-report	—	✓	✓	✓	—	Very good
Carter et al [24]	United Kingdom	My Meal Mate	NR	Self-report	—	✓	✓	—	—	Inadequate
Chen et al [34]	Australia	MyFitnessPal	Yes	Self-report	—	✓	✓	—	—	Adequate
Chmurzynska et al [37]	Poland	NR	NR	Self-report	—	✓	✓	—	—	Adequate
Costello et al [56]	United Kingdom	N/A	N/A	Active objective	—	✓	✓	✓	—	Adequate
Delisle Nyström et al [54]	Sweden	N/A	N/A	Active objective	—	✓	✓	✓	—	Adequate
Fallaize et al [32]	United Kingdom	Samsung Health; MyFitnessPal; FatSecret; Noom Coach; Lose it!	Yes	Self-report	—	✓	—	✓	—	Adequate
Griffiths et al [22]	United States	MyFitnessPal; Fitbit; Lose it!; MyPlate; Lifesum	Yes	Self-report	—	—	✓	—	—	Very good
Hezarjaribi et al [33]	United States	EZNutriPal	Yes	Self-report	—	—	✓	✓	—	Very good
Hezarjaribi et al [38]	United States	Speech2Health	No	Self-report	—	—	✓	—	—	Inadequate
Huang et al [53]	Australia	NR	NR	Active objective	—	—	—	✓	—	Very good
Hutchesson et al [45]	Australia	N/A	Yes	Self-report	—	✓	✓	—	—	Doubtful
Kato et al [62]	Japan	DialBetics	NR	Active objective	—	✓	—	✓	—	Very good
Kong et al [52]	China	N/A	N/A	Active objective	—	✓	—	✓	—	Doubtful
Lancaster et al [28]	Australia	Research Food Diary	Yes	Self-report	—	✓	—	—	—	Inadequate
Lemacks et al [31]	United States	Bridge2U	No	Self-report	—	✓	✓	—	—	Adequate
Liu et al [49]	Taiwan	NR	No	Active objective	—	—	✓	—	—	Very good
Liu et al [30]	Taiwan	NR	NR	Self-report	—	✓	—	✓	—	Adequate
Martin et al [50]	United States	NR	No	Active objective	✓	—	—	✓	—	Very good
Martin et al [51]	United States	NR	No	Active objective	✓	✓	—	✓	—	Inadequate
Most et al [63]	United States	SmartIntake	Yes	Self-report	—	—	—	✓	—	Very good
Nicklas et al [48]	United States	NR	N/A	Active objective	—	✓	—	✓	—	Adequate
Pendergast et al [21]	Australia	FoodNow	NR	Self-report	—	✓	✓	—	—	Adequate
Prinz et al [60]	Germany	N/A	N/A	Active objective	—	✓	—	✓	—	Very good
Rangan et al [20]	Australia	e-DIA^g^	NR	Self-report	—	✓	✓	—	—	Adequate
Rangan et al [19]	Australia	e-DIA	NR	Self-report	—	✓	✓	—	—	Adequate
Rhyner et al [47]	Switzerland	GoCARB	NR	Active objective	—	✓	—	—	—	Doubtful
Rodder et al [35]	United States	MyNetDiary	Yes	Self-report	—	—	✓	—	—	Very good
Rollo et al [59]	Australia	Nutricam dietary assessment method	NR	Self-report	—	✓	✓	—	—	Very good
Rollo et al [61]	Australia	Nutricam dietary assessment method	NR	Active objective	—	—	✓	✓	—	Very good
Schiel et al [64]	Germany	DiaTrace	Yes	Active objective	—	—	✓	—	—	Inadequate
Schiel et al [65]	Germany	DiaTrace	Yes	Active objective	—	—	✓	—	—	Inadequate
Smith et al [44]	China	SA-24R^h^	NR	Self-report	—	✓	✓	—	—	Adequate
Swendeman et al [43]	United States	Ohmage	No	Self-report	—	—	✓	✓	—	Very good
Teixeira et al [18]	Brazil	MyFitnessPal	NR	Self-report	—	✓	—	—	—	Doubtful
Wellard-Cole et al [29]	Australia	Eat and Track app	NR	Self-report	—	✓	✓	—	—	Very good
Zhang et al [46]	United States	Snap-n-Eat	No	Active objective	—	—	—	✓	—	Very good
Zhu et al [58]	United States	NR	NR	Self-report	—	—	—	—	✓	Very good

^a^Measurement property was either not assessed or not reported.

^b^NR: not reported.

^c^e-12HR: electronic 12-hour dietary recall.

^d^N/A: not applicable.

^e^devilSPARC: Social impact of Physical Activity and Nutrition in College.

^f^e-CA: electronic carnet alimentaire (“food record” in French).

^g^e-DIA: Electronic Dietary Intake Assessment.

^h^SA-24R: Smartphone Assisted 24 Hour Recall.

#### Self-report

##### Overview

Of the 28 studies that examined self-report methods for recording diet, 24 (86%) investigated food diary apps, 2 (7%) used ecological momentary assessment (EMA), 1 (4%) examined a smartphone-assisted 24-hour dietary recall tool, and 1 (4%) investigated the use of a web-based food database via a smartphone.

##### Food Diary Apps

A total of 24 studies investigated food diary apps [18-40,86] designed to facilitate daily or real-time recording of dietary intake. Usually, these are linked to a large database containing preprogrammed information about the energy and nutrient content of popular foods. These apps allow users to select food and beverages they have consumed, and their energy and nutrient intake for the day is automatically calculated. A wide range of food diary apps were examined within the included studies, 12 of which (described across 9 studies) [18,21,22,26,32,34,35,40,87] were publicly available on the leading app stores (Google Play or iOS).

A total of 3 studies [18,34,40] exclusively examined the measurement properties of MyFitnessPal, a widely used commercially available app, and 2 studies examined MyFitnessPal along with another app [22,32]. Furthermore, 80% (4/5) of these studies found evidence to support the validity of MyFitnessPal. Teixeira et al [18] compared the energy intake generated by MyFitnessPal with estimates generated by a paper-based food record. Griffiths et al [22] compared the app with estimates generated by a dietary analysis program [87], and Ahmed et al [40] and Fallaize et al [32] compared the app with weighed food records. They found correlations between the energy intake estimated by MyFitnessPal and their comparison measure of 0.70-0.99. Falliaze et al [32], Griffiths et al [22], and Ahmed et al [40] found no significant differences among the estimation of energy and most nutrients; however, where differences did exist, MyFitnessPal was found to yield lower intakes. Chen et al [34], by contrast, found poor agreement between MyFitnessPal and energy intake estimated via a 24-hour recall measure, finding weak to moderate correlations (0.21-0.42) and significantly lower values for total energy and all macronutrients recorded via MyFitnessPal. They found no proportional bias for energy or any of the nutrients assessed; however, wide limits of agreement were observed.

Furthermore, 2 studies investigated top nutrition tracking apps, including Fitbit, Lose it!, MyPlate, Lifesum, Samsung Health, Fatsecret, Noom Coach, and MyFitnessPal [22,32]. Both studies found strong correlations among energy and nutrient estimations via the apps and their comparison measures (0.73-0.96 and 0.79-0.91, respectively). However, numerous significant differences among nutrient estimations generated by the apps and comparison measures were identified, particularly within the *Lose it!* app. Other publicly available nutrition apps were investigated in 2 studies, with moderate mean correlations between apps and their comparison measures found (mean 0.61, SD 0.11 [26] and mean 0.67, SD 0.14 [35]).

A total of 3 studies [21,33,38] investigated the use of unstructured data entry methods to self-report food intake, compared with structured forms of recording food intake information. The unstructured data entry methods examined information about food intake recorded via free-form speech and text descriptions. Food intake information was then extracted using manual coding or natural language processing (NLP) software. Pendergast et al [21] investigated the FoodNow app, which allowed diet information to be recorded via text descriptions, voice messages, and optional images. This unstructured data were coded by trained nutritionists to match each food or beverage item described in the app to an appropriate item in a food and nutrient database [88]. They compared this approach to energy expenditure measured via the SenseWear Armband. Bland–Altman plots showed wide limits of agreement, indicating error at the individual level but no evidence of systematic bias among methods. The correlation among methods was strong (0.75), and an acceptable level of reliability among methods was found (intraclass correlation coefficient 0.75, 95% CI 0.61-0.84). Hezarjaribi et al [33,38] examined *EZNutriPal* and *Speech2Health*, 2 interactive diet monitoring systems that facilitate the collection of speech recordings and free-text data regarding dietary intake, real-time prompting, and personalized nutrition monitoring. In contrast to Pendergast et al [21] and the FoodNow app, the EZNutriPal and Speech2Health apps feature an NLP unit that allows automatic identification of food items described in the unstructured data provided. In the Speech2Health system, Hezarjaribi et al [38] used standard NLP techniques in combination with a bespoke pattern mapping technique to extract food names and portion sizes from spoken text. These data were then used to estimate the nutrient information. In EZNutriPal, Hezarjaribi et al [33] used an NLP framework based on named-entity recognition, where unrecognized entities were added to a training set to continuously update the ability of the NLP framework to correctly identify food items from an individual’s speech. Individual recognized entities relating to food items, units, and quantities were then further processed to obtain an estimate of nutrient information. This methodology was tested using 13 participants across a 13-day period using EZNutriPal. The authors found that compared with labeling of the unstructured data by patients, EZNutriPal achieved an accuracy of 89.7% in calorie intake estimation [33], whereas Speech2Health achieved an accuracy of 92.2%. In their 2019 study, Hezarjabi et al [33,38] also compared the performance of these 2 apps and found that the Speech2Health app identified 3.4 times more than the actual number of food items contained in test sentences, whereas EZNutriPal identified 0.8 times less than the actual number of food items contained in test sentences. An interesting aspect of the 2019 study of Hezarjabi et al [33] was that it explicitly incorporated personalization of the food recognition system (from voice) by allowing users to provide labels for unrecognized voice inputs. These inputs were then used to further train the algorithm and thus improve the future performance of the app.

##### EMA Apps

EMA aims to maximize the ecological validity of data collected by repeatedly collecting information about the current behaviors of participants in real-time in their natural environment [41]. Overall, 2 studies investigated apps using EMA where participants were prompted multiple times throughout the day to record their food intake [42,43]. Bruening et al [42] compared smartphone-based EMAs with 24-hour dietary recalls, whereas Swendeman et al [43] examined the agreement among EMAs of self-reported diet quality and brief dietary recall measures, anthropometric measurements, and bloodspot biomarkers. Bruening et al [42] found good agreement between their methods, with 87% of food reported in both systems. Similarly, Swendeman et al [43] found that self-reported diet quality assessed via EMAs was moderately correlated with dietary recall measures for foods with high sugar content and fast food but weakly correlated with fruits and vegetables, anthropometric, and biomarker measures.

##### 24-Hour Dietary Recall

One study investigated the performance of a smartphone-assisted 24-hour dietary recall tool in measuring beverage intake among young Chinese adults [44], comparing it with a paper-based tool and 24-hour urine samples. Participants reported significantly reduced beverage intake via the smartphone-assisted 24-hour recall compared with that via the paper-based recall and fluid intake as assessed by the smartphone, and urine volume was moderately correlated (0.58). In addition, they found evidence of systematic measurement errors whereby the bias for smartphone and paper-based recall methods were not consistent across levels of intake, with the bias increasing with higher intake of beverages.

##### Web-Based Food Database

One study [45] evaluated the accuracy of 7-day food record methods accessed on the web via a smartphone, via a computer, and using pen and paper. They found no significant differences among total energy expenditure and energy intake reported for the 3 different methods; however, their examination of the measurement error of these approaches suggested that there may be greater underreporting of energy intake using paper-based diaries compared with computer- and smartphone-based methods.

#### Active Objective

##### Overview

A total of 20 studies [46-65] examined apps that actively and objectively measured dietary intake. All studies used images of food to be consumed (and often also food waste) captured by the camera of a smartphone. Overall, 75% (15/20) of studies [48,50-52,54-57,59-65] investigated *manually analyzed food photography* methods where participants took photos of their food, which were then sent to researchers for analysis. Furthermore, 25% (5/20) of studies [46,47,49,53,58] used *automatically analyzed food photography* methods where images of food were captured by participants using specialized apps, which then analyzed images and calculated the energy and nutrient content of foods pictured automatically.

##### Manually Analyzed Food Photography

A total of 15 studies [48,50-52,54-57,59-65] used this method, of which 87% (13/15) demonstrated some evidence of its reliability and validity. Rollo et al [59,61], for example, conducted 2 studies to examine the performance of their Nutricam Dietary Assessment Method (NuDAM). NuDAM is an app that allows users to capture a photograph of food items before consumption and store a voice recording to explain the image contents before it is sent to a website for analysis by a dietitian. In their 2011 study [59], energy intake measured by the app was compared with a written food diary. Individual differences in energy intake between the 2 records varied from 6.7% to 29.7%, and on average, energy intake was underrecorded using the app. In their 2015 study [61], energy intake assessed via NuDAM was compared with weighed food records and energy expenditure using the doubly labeled water method. Moderate to strong correlations between NuDAM and weighed food records were found for energy and nutrient intakes (0.57-0.85), and mean nutrient intakes were not significantly different. The overall mean energy intake calculated by the app and weighed food records were both significantly lower than the total energy expenditure calculated using the doubly labeled water method. Participants who were found to underreport using the app were also underreported via weighed food records.

Another 6 studies [50-52,56,60,62] compared manually analyzed food photography to weighed food records and found strong correlations among methods for energy (0.92-0.99) [56,60], carbohydrates (0.93-0.99), fat (0.84-0.99), and protein (0.94-0.99) estimates [52,60]. A total of 2 studies by Martin et al [50,51] also examined the reliability of food photography methods over time and found that the energy intake estimated using this method was reliable over 3 [50] and 6 days of testing [51]. Although there was good agreement among the methods for daily energy and macronutrient intakes in Kong et al [52] and Kato et al [62], in the study by Kong et al [52], as intake increased, underestimation by the app was identified, whereas Kato et al [62] found that images captured via the app generated higher values than the weighed food record for some macronutrients. Costello et al [56] also found evidence of a small standardized bias.

In addition, 2 studies [57,63] conducted among pregnant women generated limited evidence for the validity of food photography among this population. For example, Ashman et al [57] found moderate to strong correlations among food photography and 24-hour recall for energy and macronutrients (0.58-0.84) among this population. Three studies among children and adolescents found no significant differences among energy intake estimated via food images and self-reported energy intake [64,65] or energy intake estimated via the doubly labeled water method [54]. However, in their study of 3- to 5-year-old children, Niklas et al [48] found the remote food photography method to significantly underestimate the mean daily energy intake when using the doubly labeled water method.

Similarly, Boushey et al [55] found only moderate correlations (0.58) among dietary intake estimates using the doubly labeled water method and manually analyzed food photography. There was no evidence of a systematic bias. Energy intake calculated via their app was found to be significantly less than the estimates calculated via the doubly labeled water method, with differences more pronounced in men than in women.

##### Automatically Analyzed Food Photography

A total of 5 studies [46,47,49,53,58] used this method, all of which provided some evidence of its reliability or validity. In the study by Zhu et al [58], for example, images of meals captured using a smartphone camera were segmented and identified, and their volume was estimated. *Before* and *after* images were used to estimate food intake and determine energy and nutrients consumed. The app accurately identified between 84% and 96% of 19 different food items. The study also explored the estimation of volume using 7 food items and the estimation of weight using 2 food items. The mean percentage error of the volume estimates was 5.65%. To estimate the mass, the system had a percentage error between 3% and 56%.

Liu et al [49] examined two new methods to assist with the automatic analysis of food photography—an interactive photo interface (IPI) and a sketching-based interface (SBI). The IPI presented users with images of predetermined portion sizes of a specific food and allowed users to scan and select the most representative image matching the food that they were measuring. The SBI required users to relate the food shape to a readily available comparator (eg, credit card) and scribble to shade in the appropriate area. These were compared with traditional life-sized photos commonly used by dietitians to help people identify portion sizes. The overall accuracies of the IPI, SBI, and traditional life size photo method were 66.98%, 46.05%, and 72.06%, respectively, showing that the SBI method was significantly less accurate than the IPI and traditional life size photo methods. In another study [47] investigating the GoCARB app, participants were required to place a reference card next to their plate and take 2 images using a smartphone. A series of computer vision modules detected the plate and automatically segmented and recognized different food items into 9 broad food classes (pasta, potatoes, meat, breaded items, rice, green salad or vegetables, mashed potatoes, carrots, and beans) while their 3D shape was reconstructed. The carbohydrate content of foods was then calculated by combining the volume of each food item with the nutritional information provided by a nutrition database. GoCARB estimates were compared with participant estimates of carbohydrate content and the ground truth (measured by weighing the meals and calculating carbohydrates using the same nutrition database). The mean relative error in carbohydrate estimation was 54.8% (SD 72.3%) for the estimations of participants and 26.2% (SD 18.7%) for the GoCARB app.

### Alcohol

#### Overview

A total of 16 papers examined the measurement properties of a smartphone-based approach to assess alcohol use (Multimedia Appendix 2; Table 3 for full study details). A total of 1453 participants were included in these 16 studies (range 0-671; age range 16-74 years; 510/1453, 35.09% female). Moreover, 62% (10/16) of these studies described self-report approaches, 2% (2/16) described active objective approaches, and 25% (4/16) described passive objective approaches to measuring alcohol use. The most commonly assessed measurement properties were criterion and construct validity. Although numerous apps measuring alcohol use are described here, only 1 app (Intellidrink [66]) is currently accessible via the leading app stores for consumers to monitor their own alcohol use (3 other apps [67,68,89], although publicly available, are only available for use by researchers for data collection).

**Table 3:** Key characteristics of studies examining the measurement of alcohol via a smartphone.

Study	Country	App name	Publicly available	Measurement approach	Measurement properties assessed						
					Reliability	Measurement error	Content validity	Construct validity	Criterion validity	Responsiveness	Risk of bias
Arnold et al [3]	United States	AlcoGait	NR^a^	Passive objective	—^b^	—	—	—	✓	✓	Very good
Bae et al [67]	United States	AWARE	Yes	Passive objective	—	—	—	✓	—	✓	Very good
Barrio et al [74]	Spain	SIDEAL	No	Self-report	—	—	—	✓	—	—	Doubtful
Bernhardt et al [90]	United States	HAND	NR	Self-report	—	—	—	✓	—	—	Very good
Dulin et al [72]	United States	LBMI-A^c^	NR	Self-report	—	✓	—	✓	—	—	Adequate
Kim et al [76]	United States	SPAQ^d^	NR	Active objective	✓	✓	—	—	—	—	Inadequate
Kizakevich et al [73]	United States	PHIT^e^ for duty	No	Self-report	—	—	—	✓	—	—	Very good
Luczak et al [66]	United States	Intellidrink	Yes	Self-report	—	—	—	✓	—	—	Very good
Matsumuraet al [77]	Japan	Spiral	NR	Active objective	✓	—	—	—	—	✓	Very good
McAfee et al [78]	United States	AlcoGait and AlcoWear Smartwatch app	N/A^f^	Passive objective	—	—	—	✓	—	✓	Doubtful
Monk et al [71]	United Kingdom	NR	NR	Self-report	—	—	—	✓	—	—	Very good
Paolillo et al [70]	United States	NR	N/A	Self-report	—	—	✓	✓	—	—	Very good
Poulton et al [89]	Australia	CNLab-A	Yes	Self-report	—	—	—	✓	—	—	Adequate
Santani et al [79]	Switzerland	Sensor logger and Drink logger	No	Passive objective	—	—	—	✓	—	✓	Doubtful
Swendeman et al [68]	United States	Ohmage	Yes	Self-report	—	✓	—	✓	—	—	Doubtful
Wray et al [69]	United States	Metricwire	No	Self-report	—	—	—	✓	—	—	Adequate

^a^NR: not reported.

^b^No reporting of measurement property assessed.

^c^LBMIA: Location-Based Monitoring and Intervention for Alcohol Use Disorders.

^d^SPAQ: Smartphone Addiction Questionnaire.

^e^PHIT: Personal Health Intervention Toolkit.

^f^N/A: not applicable.

#### Self-report

##### Overview

Overall, 62.5% studies [66,68-74,89,90] examined the measurement properties of apps that asked users to self-report alcohol use. Most studies (7/10, 70%) asked participants to report alcoholic beverage consumption daily [68,72,90] or in real-time (ie, using EMA) [70,71,74,89] using an app. Furthermore, 2 studies [69,73] examined the validity and reliability of completing standardized measures of alcohol use disorder via a smartphone, and 1 study [66] examined the ability of self-reported alcohol consumption via a commercially available app to accurately estimate breath alcohol concentrations (BrACs).

##### Daily Self-report

Recording alcohol consumption once a day via a smartphone app was investigated in 3 studies [68,72,90]. These studies demonstrated that this approach possesses good convergent validity when compared with traditional recall methods such as the Timeline Follow Back (TLFB) [91], which is a calendar-prompted, retrospective measure of alcohol consumption. Swendeman et al [68] found a moderate correlation (0.65) between daily self-reports and web-based 14-day recall surveys of alcohol use. However, this study also found significant differences in the mean percentage of days of alcohol use, with higher reports via daily app-based self-reports compared with 14-day recall. Similarly, Dulin et al [72] found moderate to strong correlations between data recorded through an app they tested and the TLFB for percentage of days abstinent (0.76-0.92), percentage of heavy drinking days (0.49-0.74), and the number of drinks consumed per drinking day (0.49-0.74). However, these correlations were found to diminish as more time elapsed between consumption and recall.

##### EMA Apps

A total of 4 studies [70,71,74,89] examined the measurement properties of self-reported alcohol use recorded via a smartphone using EMA, that is, as it occurred in real time or close to real time. Each of these studies employed smartphone apps that asked participants if they had consumed alcohol since the last prompt or last submission of data and the quantity consumed. Participants were often instructed to record their alcohol use as it occurred; however, Paolillo et al [70], Poulton et al [89], and Monk et al [71] also proactively prompted participants to report their alcohol use multiple times each day. These studies each demonstrated real-time, self-reports of alcohol use via a smartphone to have some convergence with retrospective reports of alcohol use, particularly the TLFB (correlations of 0.42-0.95). However, Monk et al [71] also found that participants reported consuming more drinks when reporting in real time compared with retrospective reporting. In addition, Monk et al [71] found that more drinks consumed were related to higher discrepancies between real-time and retrospective reports. Poulton et al [89] also found that participants reported a significantly faster rate of consumption when recording in real time via an app, compared with retrospective accounts.

##### Standardized Measures of Alcohol Use Disorders

A total of 2 studies [69,73] examined the measurement properties of administering the Alcohol Use Disorders Identification Test (AUDIT), a standard measure of alcohol use with established reliability and validity, via a smartphone [75]. In their study, Kizakevich et al [73] compared the AUDIT completed via their app with pen and paper administration of the measure. Wray et al [69] asked participants to complete the AUDIT once a day for 30 days and compared this with the TLFB. Both studies provided some evidence for the validity of completing the AUDIT via a smartphone app. Kizakevich et al [73] demonstrated that there was very good convergence between the AUDIT completed on paper and via the app (0.97), whereas Wray et al [69] found that the AUDIT and web-based TLFB were moderately correlated (0.55-0.88). Wray et al [69] found evidence of underreporting alcohol use on the TLFB.

##### BrAC Apps

Luczak et al [66] investigated the ability of a transdermal alcohol concentration (TAC) device in combination with a commercially available app *Intellidrink* to estimate BrAC. TAC devices measure the amount of alcohol diffusing through the skin at a particular time. As the raw TAC data are not directly related to blood alcohol concentration (BAC) or BrAC, further information on consumed alcoholic drinks is required to calibrate the models that convert TAC data to BrAC. The Intellidrink app was used to allow participants to self-report basic demographic data and data for each drinking episode. These data were combined with the TAC data in the authors’ BrAC estimator software to accurately estimate BrAC. The authors found that the BrAC algorithm combined with the Intellidrink app had good convergent validity when compared with results generated by the previously validated breath alcohol estimator software developed by the authors. The combination of TAC device and Intellidrink app calculated peak BrAC estimates (eBrAC) to within 0.0003% of that calculated by the BrAC estimator software when using raw breadth analyzer data. The Intellidrink calculated time of peak eBrAC was within 18 minutes of the reference data, and the area under the eBrAC curve was within 0.025% for alcohol hours.

#### Active Objective

Two studies [76,77] investigated approaches to actively and objectively measure alcohol use via smartphones. One [77] examined the potential of a mobile-based test of psychomotor performance to measure alcohol-induced impairment, whereas the other [76] described the validation of an optical attachment for smartphones to identify the results of saliva alcohol concentration test strips.

In their study, Matsumura et al [77] tested the performance of participants on a mobile-based test of psychomotor performance (Spiral for iPhone) and 3 computer-based tests assessing psychomotor and cognitive performance at predrink baseline (BAC of 0%) and after alcohol consumption. When participants had a BAC close to 0.1%, their performance on all tests, including the Spiral for iPhone, was found to be significantly worse than baseline and 0% BACs. Although significant decreases in performance accuracy for the 3 computer-based tests were also found when participants had BACs close to 0.06%, performance on the smartphone-based test (Spiral for iPhone) was not significantly worse than baseline.

Kim et al [76] examined a custom-built smartphone attachment and smartphone app to capture an image of saliva alcohol concentration test strips and identify their correct saliva alcohol concentration. Using their system, they inserted test strips into the custom-built smartphone attachment, and images were captured using the camera of the smartphone. Their smartphone app used machine learning techniques to calculate the estimated saliva alcohol concentration. The authors used test strips prepared with various concentrations of ethyl alcohol to generate the training data. A total of 14 images were recorded for each concentration, but the study by Kim et al [76] did not report how many of these were used for training, and how many were used for unbiased testing of the trained machine learning algorithms. The authors reported that this approach to analyzing saliva alcohol concentrations is valid and reliable across different types of smartphones, providing average classification rates of 100% accuracy for standard concentrations (0%, 0.02%, 0.04%, 0.08%, and 0.3%) and 80% accuracy for intermediate concentrations that required finer discrimination.

#### Passive Objective

##### Overview

A total of 4 studies examined the measurement properties of passive objective approaches to measure alcohol use. Arnold et al [3] and McAfee et al [78] used smartphone sensors [3] and a combination of smartphone and smartwatch sensors [78] to measure gait as a proxy for alcohol-induced impairment, whereas Santani et al [79] and Bae et al [67] used phone sensor data to infer alcohol use (see Multimedia Appendix 2 for full study details).

##### AlcoGait App

A total of 2 studies [3,78] investigated whether a smartphone user’s level of alcohol intoxication could be accurately inferred from their gait. Both studies used the AlcoGait app, which runs continuously in the background of smartphones of users. In the study by Arnold et al [3], accelerometer data were collected by the app, and information about users’ gait generated. This information was then labeled the following day using an in-app survey that asked users to identify when they began drinking and finished drinking and how many drinks they had. Machine learning algorithms were trained with these data to infer BAC as membership of one of the three classes: 0 to 2 drinks, 3 to 6 drinks, or >6 drinks. In their study, McAfee et al [78] extended the AlcoGait app with the AlcoWatch to create the AlcoWear system, which also uses gyroscopes to capture information on the rotational velocity of the smartphone in response to the user’s movement.

Both studies generated evidence for the validity of this approach. Arnold et al [3] found that after training the system on 209 data points, the AlcoGait app could classify the alcohol consumption of a user into 1 of the 3 classes with an accuracy of 56% (*F* score of 0.629 and area under the receiver operating characteristic curve [AROC] of 0.685) on training data. They reported a higher performance of 70% (*F* score of 0.786 and AROC of 0.825) on yet unseen data. McAfee et al [78] used 33 participants wearing sensor-impairment goggles to simulate the effects of alcohol consumption on the body. Training data were gathered by extracting features such as step count, cadence, and sway from 90-second walks with sensor-impairment goggles simulating BAC in 4 ranges (0.04-0.06, 0.08-0.15, 0.15-0.25, and 0.25-0.35). These training data were then used to train and validate several machine learning algorithms. They found that the AlcoGait app was able to infer the correct BAC range with an accuracy of 89.45% with 99% of the data used for training and 1% used for validation. The authors reported a maximum accuracy of 79.8% when using the smart watch to infer BAC as being higher or lower than 0.08.

##### Smartphone Sensors

Overall, 2 studies [67,79] examined the use of data from multiple smartphone sensors and machine learning to automatically recognize drinking behavior. They both used apps that run in the background on user’s phones to collect sensor data from participants’ phones. Bae et al [67] used the app AWARE to collect data continuously over 28 days from 38 young adults with hazardous drinking. They collected information relating to time (eg, day of week or time of day), movement (eg, accelerometer or gyroscope), communication (phone calls or texts), and psychomotor impairment (keystroke speed; available for Android phones only) and used these data to train random forests to predict periods of no drinking, low-risk drinking, and high-risk drinking from historic data (1- and 3-day history). Alcohol use information was collected via SMS text messages sent at 10 AM each day asking about the previous day. The performance of their algorithms was tested using 20% of the data not previously used for training.

Santani et al [79] used the Android app SensorLogger to collect information related to location (GPS or Wi-Fi), movement (accelerometer), social context (density of nearby Bluetooth devices), and phone use (battery, screen, and app use) on 10 weekend nights from 8 PM to 4 AM from 241 participants. The DrinkLogger app was then used to allow participants to report their alcohol consumption when it occurred. After preprocessing, 1011 user nights from 160 individuals were used to train a random forest algorithm with 500 trees to predict whether a user had consumed alcohol on a given night.

Bae et al [67] provided some evidence of the validity of using mobile phone sensors and machine learning algorithms to identify alcohol use among young people. They found that drinking categories were significantly correlated with time of day (0.11) and day of week (0.06), claiming that with time of day and day of week alone, they were able to detect low- and high-risk drinking with 90% accuracy. Their best-performing model to predict drinking used random forests and 3 days of historical data from multiple sensors. The model had a Cohen κ of 0.80 and an AROC of 0.96 and correctly classified 30-minute windows of time as nondrinking 98.5% of the time, low-risk drinking 70.2% of the time, and high-risk drinking 84.4% of the time. The AROC is a measure of the ability of an algorithm to achieve high sensitivity as well as high specificity and has a maximum value of 1, indicating a perfect classifier. Random predictions (of drinking in this case) would result in an AROC score of 0.5. In contrast, Santani et al [79] found that even data from their most informative features (accelerometer data) could only identify drinking nights with 75.8% accuracy. This was followed by location, Wi-Fi, and Bluetooth logs with 68.5%, 65.2%, and 64.2% accuracy, respectively (note that a random guess would have resulted in an accuracy of 67%, as 67% of the data used reported alcohol consumption for that night).

### Tobacco

#### Overview

A total of 8 studies [68,80-85,92] described the measurement properties of approaches assessing tobacco use. The number of participants involved in these studies ranged from 3 to 146 (N=363). The studies involved, on average, 34.7% female participants ranging in age from 18 to 64 years. The key characteristics of the 8 studies are shown in Table 4 (see Multimedia Appendix 2 for full study details). Furthermore, 50% (4/8) of the studies described active objective approaches to measure smoking, 12% (1/8) used a self-report method, and 37% (3/8) described passive objective approaches. All 8 studies assessed the construct validity (specifically convergent validity) of their approaches. Although several different apps are described in the included studies, only 4 apps are accessible via the leading app stores (Instant Heart Rate, Cardio [83], Smokerlyzer [82], and SmokeBeat [84]), and designed to help consumers monitor their own tobacco use.

**Table 4:** Key characteristics of studies examining the measurement of tobacco via a smartphone.

Study	Country	App name	Publicly available	Measurement approach	Measurement properties assessed					
					Reliability	Measurement error	Construct validity	Criterion validity	Responsiveness	Risk of bias
Dar [84]	Israel	SmokeBeat	Yes	Passive objective	—^a^	—	✓	—	—	Doubtful
Herbec et al [83]	United Kingdom	*Instant Heart Rate* or *Cardio*	Yes	Active objective	—	—	✓	—	✓	Doubtful
McClure et al [80]	United States	My Mobile Monitor	No	Active objective	—	—	✓	—	—	Doubtful
Meredith et al [81]	United States	NR^b^	NR	Passive objective	✓	—	✓	✓	✓	Doubtful
Qin et al [92]	Canada	NR	NR	Passive objective	—	—	✓	—	—	Doubtful
Shoaib et al [85]	Netherlands	NR	No	Passive objective	—	—	✓	—	—	Very good
Swendeman et al [68]	United States	Ohmage	Yes	Self-report	—	✓	✓	—	—	Doubtful
Wong et al [82]	Malaysia	Smokerlyzer and iCOSmokerlyzer	Yes	Active objective	✓	—	✓	✓	—	Doubtful

^a^No reporting of measurement property assessed.

^b^NR: not reported.

#### Self-report

One study [68] examined the measurement properties of a self-report method to assess tobacco use. In this study, HIV-positive adults were asked to complete daily mobile surveys when prompted by the app, and whenever they smoked, for 6 weeks. Participants were asked to indicate if they had smoked since the last time they self-reported via the app. This study demonstrated that there was very good convergent validity among daily mobile self-reports and web-based 14-day recall surveys of tobacco use, with a strong correlation between methods (0.92).

#### Active Objective

##### Overview

Of the 4 studies that examined active objective measures of tobacco use, 3 (75%) [80-82] investigated the measurement of expired CO using a smartphone app in conjunction with expired CO monitors, and 1 (25%) [83] investigated whether heart rate measured by a smartphone could accurately identify smoking episodes.

##### Expired CO

A total of 3 studies used this methodology [80-82], and all found evidence to support its validity and reliability. Overall, 67% (2/3) of these studies [81,82] used expired CO monitors designed to attach directly to smartphone users. Meredith et al [81] described the use of a prototype CO monitor for smartphones, developed by the authors, and Wong et al [82] examined the first commercially available CO monitor for use with a smartphone and accompanying smartphone app (iCO Smokerlyzer and the Smokerlyzer app). Both studies found that the first and second CO measures collected via their smartphone CO monitors were strongly and significantly correlated with each other (0.98 and 0.94, respectively). Both studies also found that measurements of expired CO collected via smartphone-attached CO monitors were strongly correlated with measurements collected via stand-alone CO monitors. A third study [80] described a protocol whereby young smokers (aged 15-25 years) used a smartphone app (MyMobile Monitor) and the camera of their smartphone to take time-stamped photographs of themselves exhaling into a stand-alone expired CO monitor (PiCo Smokerlyzer), and a photograph of the CO readings displayed by the monitor to be verified by the research staff. This study found a moderate agreement among the methods (0.49).

##### Heart Rate Apps

One study [83] used 2 publicly available heart rate apps (*Instant Heart Rate* and *Cardio*) to investigate whether resting heart rate measured using a smartphone could be used to verify smoking abstinence. The study by Herbec et al [83] of 18 adult daily smokers found some evidence to support this approach. Specifically, they found that lower heart rates were observed among all participants on days they did not smoke and did not use nicotine replacement products, compared with days on which they smoked as usual. Similarly, lower heart rates were also observed among 83% (15/18) of participants on days they were abstinent but used a nicotine replacement product compared with those on days when they smoked as usual.

#### Passive Objective

##### Overview

A total of 3 studies [84,85,92] investigated passive objective approaches to measure tobacco use via smartphones. Overall, 67% (2/3) of these studies [84,85] used wrist-worn sensor devices (eg, smartwatches) in conjunction with smartphone apps to detect episodes of smoking. A third [92] used in-phone sensors only to recognize the occurrence of smoking might be taking place.

##### Wrist-Worn Sensors

Shoaib et al [85] used accelerometer and gyroscope data collected from smartwatches and smartphones to test a 2-layer hierarchical smoking detection algorithm. In their study, participants wore a smartwatch on their right wrist and a smartphone in their right pocket. A total of 11 participants performed 17 hours (230 cigarettes) of smoking while sitting, standing, walking, and in group conversation and 28 hours of other similar activities (eg, eating and drinking). Data were collected at 50 samples per second from these sensors. Dar [84] provided participants with smartwatches and instructed them to wear them on the hand that they used for smoking. Dar [84] then used the SmokeBeat app to process raw data from these devices, identify smoking episodes, and provide feedback to participants in real time. These 2 studies demonstrated very good convergent validity with self-reported smoking episodes. Shoaib et al [85] achieved a very high precision and recall for smoking in 83% to 97% *F*-measure, whereas Dar [84] detected 82.29% of smoking episodes, with a negligible frequency of erroneously detected episodes (2.85%).

##### In-Phone Sensors

One study [92] used data collected from the GPS, Wi-Fi, and accelerometer within the smartphones of participants and self-reported smoking behaviors, collected over 1 month to train and evaluate algorithms to accurately classify smoking and nonsmoking periods based on in-phone sensor data alone. First, each of the individual features extracted from the sensor data collected was used to train univariate hidden Markov models (HMMs), which were then evaluated. Next, multivariate HMMs using 3 features and 5 features were trained and evaluated. Qin et al [92] were able to detect smoking activity with an accuracy over 0.9, and an AROC of >0.8. HMMs with a single feature were found less accurate than multivariate HMMs.

### Risk of Bias

Most studies (35/48, 73%) investigating the measurement of diet received *very good* (23/48, 48%) or *adequate* (12/48, 25%) methodological quality ratings on the COSMIN Risk of Bias tool (Table 2). As Table 3 shows, most studies (11/16, 69%) investigating the measurement of alcohol use were of at least adequate methodological quality (*very good*, 8/16, 50%; and *adequate*, 3/16, 19%), whereas only 13% (1/8) of the studies investigating the measurement of tobacco use received a *very good* rating (Table 4).

Across the 3 behaviors, 73% (29/40) of studies investigating self-report measurement approaches received *very good* or *adequate* quality ratings. This is compared with 60% (15/25) of studies investigating active objective approaches and 38% (3/8) of studies investigating passive objective approaches. Ratings of *adequate* or *very good* were achieved by 72% (21/40) of diet self-report studies, 74% (14/19) diet active objective studies, and 63% (5/8) alcohol self-report studies. By contrast, *adequate* or *very good* ratings were achieved by only 50% (1/2) of alcohol active objective and 50% (2/4) alcohol passive objective studies, by 25% (1/4) of tobacco passive objective studies, and by none of the tobacco self-report and active objective studies.

The 3 most commonly investigated measurement properties were also found to be the most rigorously examined. Overall, 71% (22/31) of studies examining measurement error, 96% (26/27) of studies examining criterion validity, and 76% (35/46) of studies examining construct validity received very good or adequate ratings for their examination of these measurement properties. In contrast, reliability and responsiveness were only examined with very good or adequate methodological quality in 29% (2/7) and 40% (2/5) of the studies, respectively.

## Discussion

### Principal Findings

#### Overview

This systematic review is the first to bring together the existing evidence of the measurement properties of smartphone-based approaches to measure three key lifestyle behaviors—diet, alcohol use, and tobacco use. Overall, there was some evidence to support the reliability and validity of using smartphones to assess these behaviors. However, results varied by behavior and measurement approach, and the methodological quality of studies ranged from inadequate to very good. To an extent this large range can be attributed to the significant number of new technologies and methodologies being designed and tested for automated measurements of the behaviors of interest. These methods are not yet mature enough but may provide exciting opportunities as they develop further.

Studies most commonly focused on approaches to assess dietary behaviors (48/72, 67%), with only 22% (16/72) and 11% (8/72) measuring alcohol and tobacco use, respectively. Across the health behaviors, 19 different smartphone-based measurement techniques were used, the most commonly examined approach being food diary apps. Most studies investigated the construct validity, criterion validity, or measurement error associated with their measurement technique, whereas few examined their reliability or responsiveness. Although a wide range of smartphone apps were described in the included studies, most of these apps are not currently publicly available or are designed as research data collection tools, rather than tools that can be easily used by clinicians or consumers to monitor diet, alcohol use, or tobacco use behaviors.

The highest quality evidence was found for diet, with most studies examining diet being rated as very good or adequate (35/48, 73%). This was compared with those assessing alcohol use (11/16, 69%) and tobacco use (1/8, 13%). In light of the fewer number of studies and poorer quality of evidence available, conclusions and recommendations drawn from the existing literature regarding smartphone-based measurement of alcohol and tobacco use should be interpreted with caution.

#### Diet

Diet was most commonly examined using self-report methods. These studies indicated that food diary apps, in particular MyFitnessPal, can be a reliable and valid method of measuring energy intake. Individual studies investigating other smartphone-based self-report methods, including 24-hour recall, web-based food databases, EMA, and food diary apps using unstructured data entry methods, have demonstrated promising results. However, there is currently insufficient evidence supporting the reliability or validity of these approaches, and further research is required. A growing body of literature suggests that manually analyzed food photography may be valid and reliable in a general adult population. However, because of the need for highly trained individuals to analyze every captured image, this approach is unlikely to be scalable or sustainable outside of a research context.

This review identified a small body of literature investigating the novel approach of using smartphones to capture images and voice, extract food intake information from these data, and access external databases to retrieve nutrient information. These studies relied on spoken reports by users or on the use of machine learning to automatically recognize food items (and their size) in photographs. Although the results are encouraging, most of these studies confined their investigations to a small number of food items, with tests performed on a small number of participants. Hence, the generalizability of these results cannot be assessed. Given the large variation in the appearance of food in a global society, such approaches will likely require vast amounts of varied training data to be of general applicability. Promisingly, the lower burden this automatic analysis approach places on users and administrators and its potential to provide real-time feedback to users mean that this approach is potentially scalable and could be a powerful addition to eating behavior interventions. Both manually and automatically analyzed food photography methods address some of the key issues associated with traditional methods for measuring diet behavior. As long as users remember to take a photograph of their food, these methods provide an objective record of their food intake reduction issues associated with recall bias, and the use of fiducial markers (as was common in included studies) reduces the reliance on users to be able to accurately estimate portion sizes.

An important limitation of most of the studies that investigated the measurement of diet behaviors was small sample sizes, with 77% (37/48) of the studies involving under 100 participants, including 50% (24/48) of the studies with <50 participants.

#### Alcohol

As with diet, most studies assessing alcohol use used self-report methods. A strength of this literature is the frequent (though not universal) use of a common comparison measure, the TLFB [91]. The included studies provided good evidence for the validity of daily and real-time self-reporting of alcohol use via smartphones, with moderate to strong correlations with retrospective reports of alcohol use found across studies. However, several of these studies [68,71,72,89] also found that participants reported greater alcohol use via smartphone-based reporting compared with retrospective reports of alcohol use, such as the TLFB. These discrepancies were interpreted by a number of authors [71,72,89] as evidence that underreporting of alcohol use occurs when using recall methods and that app-based self-reports of alcohol use may be able to provide a better understanding of alcohol intake [89]. This interpretation is problematic, as the TLFB is widely acknowledged as the *gold standard* measure for self-reported alcohol use. Unfortunately, none of the included studies also used an objective measure of alcohol use, which may have elucidated this finding and would have allowed a comparison of the accuracy between app-based self-report and the TLFB.

Insufficient evidence for the smartphone administration of AUDIT [93], a standardized measure of alcohol use disorder, has been generated to date. Similarly, although the results of a study examining if BrACs could be accurately estimated via a smartphone app were positive, there is insufficient evidence at this stage supporting this approach. Two studies assessed novel active objective methods for measuring alcohol use. For example, the study by Matsumura et al [77] suggests that smartphone-based measures of psychomotor performance may be able to validate alcohol-induced impairment. However, more studies involving larger sample sizes are required before it can be determined if initial promising results are representative of the true measurement properties of these approaches.

Although only 4 studies focused on passive objective measurement of alcohol use, these findings suggest that using in-built phone sensors to infer and even predict alcohol use may be a promising assessment method. However, some methodological issues are worth noting. For example, McAfee [78] likely severely *overfit* their data by training their algorithm on 99% of their data, meaning that their approach is unlikely to be able to generalize to a new data set. Further research is needed before the validity and reliability of these types of methods can be established and will likely include the gathering of large amounts of data. To be of most use to clinicians, consumers, and researchers who are interested in passively measuring alcohol use, easy-to-use interfaces that automatically process sensor data (preferably in real time) and feedback results are needed.

A limitation of most studies that investigated measurement of alcohol use was the small sample size, with 81% (13/16) of the studies involving samples of <100 participants (11/16, 69%, with <50 participants). In addition, many included studies were conducted within specific populations, such as college students, people with HIV, and military personnel, which may limit the generalizability of results to the broader population.

#### Tobacco

Measuring tobacco use with smartphones has been examined by the fewest number of studies. Unlike other behaviors, most tobacco use studies have focused on objective measurement techniques rather than self-reports. A total of 3 studies supported the methodological soundness of measuring expired CO using smartphones (and expired CO monitors). Using apps that measure users’ heart rate was also found to be a promising way to quickly and easily verify smoking abstinence. Passive measurement approaches using wrist-worn and in-phone sensors also show promise.

Although the results from these individual studies are promising, further research is needed to establish the validity and reliability of these types of objective approaches. In addition, most studies involved very small samples (7/8, 88%, involved <100 participants; and 6/8, 75%, involved <50 participants).

### Strengths and Weaknesses of Measurement Approaches

This study identified three key approaches used to measure diet, alcohol use, and tobacco use via smartphones: self-report, active objective, and passive objective approaches. Across behaviors, several key strengths and weaknesses associated with these approaches have emerged. To date, most evidence has been generated for self-report and smartphone-based measurement approaches. These approaches are most similar to traditionally used measurement approaches and often involve simply asking users to complete existing validated measures of behaviors by interacting with the touchscreen of their smartphone, rather than completing them using a pen and paper survey or an interview. Moving self-report measures onto a smartphone, a device that many people carry with them and that can automatically calculate summary information, improves upon traditional measurement approaches by facilitating real-time recording of health behaviors and providing feedback to users—a potentially powerful intervention tool [94]. Although many self-report methods have been shown to be reliable and valid, particularly for diet, these approaches remain burdensome and require considerable input from the user. It is also likely that smartphone-based self-report measures continue to suffer from biases similar to traditional self-report systems, for example, as response bias and declining accuracy over time.

This review identified several novel approaches to objectively measure diet, alcohol use, and tobacco use, both with and without the active involvement of participants. Fewer studies that used objective approaches (active and passive) received a quality rating of *very good*, compared with self-report approaches. This perhaps is not so much a criticism of these approaches as an acknowledgment that many of these studies used innovative machine learning methods with limited data sets and require further investigation before they can be considered mature. The obvious strength of these approaches is that they have the potential to provide objective measurements of consumption behaviors, which have traditionally been primarily assessed using self-report measures. These approaches can address issues with reporting accuracy, recall bias, and memory. However, for alcohol and tobacco use, the objective, smartphone-based measurement approaches developed to date do not directly assess these behaviors (as is the case for diet with food photography methods). Rather, these approaches use proxy measures related to the physiological response to the behaviors (eg, measuring CO content or BAC, or measuring gait to infer alcohol intoxication) or infer the physical movements associated with the behaviors (eg, hand movements to infer cigarette smoking). For all 3 behaviors, the results of active objective measurements suggested that, although these methods have good potential to significantly reduce the user burden and recall bias, they can still be quite burdensome for users and may not be particularly scalable, as for manually analyzed food photography methods.

Although passive objective approaches may address the issue of participant burden by collecting information from smartphones without the involvement of users, this continuous collection and storage of sensor data from phones is associated with privacy and data security issues, which may mean that these powerful approaches are not acceptable to many people. However, previous research indicates that when employed for health purposes (eg, sharing sleep, mood, or physical activity information with a physician), most people are comfortable sharing passively sensed information, and characteristics such as age may not influence the comfort of individuals by sharing this sort of information [95]. With only 7 studies that described the measurement properties of passive objective approaches, more research is needed to establish the validity and reliability of these approaches. Although sufficient evidence may not yet exist to recommend the use of passive objective measurement approaches, these types of approaches have huge potential to augment health behavior change interventions. Information gathered in this way can potentially be used to provide tailored support *in the moment* to users, allowing relevant support to be delivered during a time and context when it is most salient [67].

### Recommendations and Future Directions

Although 72 studies were identified that aimed to describe novel smartphone-based approaches to measuring diet, alcohol use, and tobacco use, a major issue identified within this literature is the extreme heterogeneity in approaches and evaluation methods investigated. Nineteen broad measurement techniques were described in studies included in the current review, and within these groups, almost every individual study described a different specific technique. For example, each food diary app described used a different way to record diet information, used different food databases to provide nutritional information of recorded food items and different methods for entering data. Similarly, the algorithms used to automatically analyze food images or indicate that alcohol and tobacco use from sensor data differed. The relatively short time smartphones have been available (approximately 13 years) and the relatively early stage of research in this area may explain the lack of homogeneity in the types of specific techniques and methods investigated.

Noting the above limitations of current knowledge in this area, clinicians and consumers looking for valid, reliable, and publicly available ways to assess diet and alcohol use behaviors might consider using food diary apps such as *MyFitnessPal* [18,22,32,34,40] and apps that assess alcohol use via daily or real-time self-reports (eg, *Intellidrink*) [66]. Smartphone-compatible CO monitors such as the iCoSmokerlyzer and Smkerlyzer apps are also promising ways to assess tobacco use [82]; however, further research in this area in particular is needed.

Although it is important to continue moving the field forward and investigate if new and better ways to measure consumption behaviors using smartphones can be developed, it is strongly recommended that researchers first look to the existing literature described here (and in other fields) to determine if, in the search for a way to measure diet, alcohol use, or tobacco use, using a smartphone, an existing technique, or an app may be appropriate for their purposes before considering development of yet another app. Agreed-upon standards for capturing the data and extracting higher-level information (such as nutrient information) would be a constructive way of ensuring that the data collected can be pooled with similar data from other initiatives, thus providing a larger and more robust data set for algorithm development.

Only 4 studies [42,43,64,65] described apps that assessed >1 behavior (specifically diet and physical activity behaviors). Building or identifying systems that allow easy and accurate measurement of multiple health behaviors would be a useful addition to the field as we know that health risk behaviors such as poor diet, substance use, physical inactivity, and poor sleep [96].

The heterogeneity of methods used to evaluate the measurement properties of techniques is another weakness of the current literature. Again, it is recommended that researchers examine the existing literature closely when designing their own studies. For example, it is suggested that the TLFB be considered as a comparison measure for smartphone-based approaches to measure alcohol use, as it has been most frequently used in the current literature. However, no such common comparison measure of diet and tobacco use has emerged from the literature to date. In addition, the accuracy of self-reported measures of these consumption behaviors has been questioned [68,71,72,89], and it has been suggested that newer measurement approaches, such as the smartphone-based approaches discussed here, may in fact provide data closer to the actual behaviors under investigation and may eventually be themselves considered the gold standard in the measurement of these behaviors. In the meantime, it is recommended that researchers consider investigating the validity of smartphone-based approaches in comparison with objective measurements of these behaviors. Indeed, this review identifies a lack of objective comparisons as a key weakness, with few studies (particularly for alcohol and tobacco use) investigating the criterion validity of these approaches. Similarly, other measurement properties, such as reliability and responsiveness, have rarely been investigated. To take full advantage of smartphones in research, in clinical settings, and within consumers’ everyday lives, the full variety of measurement properties of these different approaches needs to be better understood.

To have the biggest impact on chronic disease, we need to make valid and reliable tools easily available to clinicians and consumers to allow for the collection of quality and detailed health behavior information. There is also a need for easy-to-use interfaces to facilitate the use of these passive sensing systems by clinicians and consumers. Quality and detailed information regarding diet, alcohol use, and tobacco use can be leveraged to help individual consumers acquire better insights into their own behaviors and inform tailored support. In other words, it is important that the apps used to measure diet, alcohol use, and tobacco use are publicly and freely available.

### Limitations

An important limitation of this review is that it included only studies published up to March 2020. In this rapidly growing area, there are likely to be recent and ongoing studies that have also investigated the measurement properties of smartphone-based approaches to measuring health behaviors. In addition, this review only captures approaches whose measurement properties have been examined and discussed in the published literature. It is likely that other novel and potentially effective approaches to measure diet, alcohol use, and tobacco use have been developed and are currently in use but that they have been developed outside of academia, their measurement properties have not been not specifically assessed, or they simply have not been published.

### Conclusions

Accurate measurement of diet, alcohol use, and tobacco use is central to successful chronic disease risk reduction interventions [8-11]. Therefore, identifying new and valid ways to measure these behaviors could have major public health implications. This review highlights measurement approaches that clinicians and researchers may want to consider implementing to help clients better measure and manage their health behaviors and improve the measurement of these behaviors in research settings. The results suggest that food diary apps, particularly the commercially available app MyFitnessPal, may be appropriate tools to measure diet. The review also highlights approaches with growing bodies of promising evidence but where more research is needed before their use might be recommended (eg, food photography methods and CO monitor smartphone attachments). Finally, the review highlights several measurement approaches with great potential but where only mixed evidence or evidence from 1 or 2 studies is available (eg, smartphone-based measurement of psychomotor performance to infer alcohol intoxication; the use of smartphone and wrist-worn sensors to infer alcohol intoxication and detect or predict alcohol and tobacco use; and the use of heart rate monitor apps to infer smoking abstinence). These conclusions should not be interpreted as a criticism of these approaches but rather as an acknowledgment that many of these approaches use cutting-edge technologies, which require further research (and data) before they can be expected to yield accurate and generalizable results.

## Appendix

Multimedia Appendix 1 MEDLINE search strategy.

Multimedia Appendix 2 Additional details and results of included studies.

## Abbreviations

AROC: area under the receiver operating characteristic curve
AUDIT: Alcohol Use Disorders Identification Test
BAC: blood alcohol concentration
BrAC: breath alcohol concentration
CO: carbon monoxide
COSMIN: Consensus-Based Standards for the Selection of Health Measurement Instruments
eBrAC: breath alcohol concentration estimates
EMA: ecological momentary assessment
HMM: hidden Markov model
IPI: interactive photo interface
NLP: natural language processing
NuDAM: Nutricam Dietary Assessment Method
PRISMA: Preferred Reporting Items for Systematic Review and Meta-Analyses
SBI: sketching-based interface
TAC: transdermal alcohol concentration
TLFB: Timeline Follow Back
