# Supplementary material for: Measurement Properties of Smartphone Approaches to Assess Diet, Alcohol Use, and Tobacco Use: Systematic Review
Source: JMIR Mhealth Uhealth. 2022 Feb 17;10(2):e27337. doi: 10.2196/27337 (PMC8895282; doi:10.2196/27337)
Supplement: Multimedia Appendix 2 [file mhealth_v10i2e27337_app2.pdf]

Multimedia Appendix 2: Additional details and results of included studies

Table of Contents

Diet - Self Report 2

Diet - Active Objective 13

Alcohol - Self-Report 22

Alcohol – Active Objective 24

Alcohol – Passive Objective 25

Tobacco - Self-report 27

Tobacco – Active Objective 27

Tobacco – Passive Objective 29

Supplementary Table 2. Additional details and results of included studies

Paper details									App/Phone Information			Comparison measure	Measurement Properties Results
First author	Year	Country	N	% Female	Mean Age	SD Age	Age Range	Other population information	App Name	Details of the app/ smartphone data collection	Operating system		
**Diet - Self Report**													
Teixera	2018	Brazil	30	73.00%	22.8	2.6	18-30	University students	MyFitnessPal	Food diary app	NR	Construct validity - mobile food record with a paper food record	Measurement error: Bland-Altman plots showed good agreement between methods, but evidence of systemic errors for lipids. Construct validity: Moderate correlations between methods for energy (r = 0.70, P < 0.01), and fibre intake (r = 0.63, P < 0.01).
Smith	2014	China	110	46.00%	29.6	0.29	20-40	24% overweight/ obese	SA-24R	Smartphone assisted 24-hr recall	Android	Criterion validity - paper based WA-24R. Construct validity - 24hr urine samples measuring total urine volume	*Measurement error:* Bland-Altman plots showed that for total beverages and for sugar sweetened beverages the bias for the two recall methods was not consistent across levels of intake, with the bias increasing with higher intakes of beverages. *Construct Validity*: Participants reported significantly fewer g/day total beverages via the smartphone assisted 24 hour recall than via the paper-based recall. Correlations between smartphone assisted and paper-based recall methods ranged between 0.21 (fruit juice) and 0.60 (total beverages), with the exception of a correlation of −0.06 for sweetened fruit drinks (this latter beverage type was the smallest contributor (<3%) to beverage intake). *Criterion validity:* Fluid intake as measured by smartphone assisted 24 hours recall and as estimated by collected urine volume was moderately correlated (0.42). Paper based 24 hour recalls were only weakly correlated with urine volume (0.31). Four individuals

													were identified with extremely poor agreement between the recall methods and urine volume. Excluding these individuals improved correlations to 0.58 for smartphone assisted recall and to 0.46 for paper-based recall.
Rangan	2016	Australia	80	62.00%	21	1.5	19 - 24		e-DIA (electronic Dietary Intake Assessment)	Food diary app	Android or iOS	Construct validity - 24-hour dietary recalls	*Measurement error:* Bland-Altman plots showed robust agreement between methods, with no evidence of systematic bias. *Construct validity:* No significant differences were found in median intakes between methods for any of the food groups. Moderate correlations between methods found, range of 0.69 to 0.88 (mean r across food types = 0.79),
Rangan	2015	Australia	80	62.00%	NR	NR	19 - 24		e-DIA (electronic Dietary Intake Assessment)	Food diary app	Android or iOS	Construct validity - 24-hour dietary recalls	*Measurement error:* Bland-Altman plots showed wide limits of agreement between the methods but without obvious bias. *Construct validity:* No significant differences between energy and nutrient intakes record by either method. Moderate correlations between methods found, range of 0.50 to 0.79 (mean r across all nutrients = 0.66).
Pendergast	2017	Australia	90	79.00%	24.9	4.1	18-30		FoodNow	Food diary app - unstructured data entry	Android or iOS	Energy expenditure measured via a SenseWear Armband	Measurement error: Bland-Altman plots showed wide limits of agreement, indicating error at an individual level, but and no evidence of systematic bias between methods. Construct validity: Correlation between methods was strong (r=0.75). A high degree of reliability was found between methods was also found (ICC, 95% CI: 0.75, 0.61, 0.84).

Hutchesson	2015	Australia	18	100.00 %	23.4	2.9	18-30		NA	Online food database accessible via smartphone	Any smartphone with internet access	Total energy expenditure measured by calorimetry and physical activity level derived from accelerometers.	*Measurement error:* Bland-Altman plots showed wide limits of agreement for all three food records, indicating inconsistencies between total energy expenditure and energy intake at the individual level. The limits of agreement were similar for smartphone, and computer, but considerably narrower for the paper-based food records, suggesting there may be more discrepancy between total energy expenditure and self-reported energy intake using paper-based diaries compared with online and smartphone based methods. *Construct validity: Underreporting of total energy expenditure was reported across all three methods.* There were no significant differences in absolute or percentage mean difference between total energy expenditure and energy intake reported for the different methods, suggesting that all three methods produced similar levels of under reporting.
Griffiths	2018	USA	30		NR	NR	NR		Top 5 nutrition tracking apps available for free download in the Apple store (as of Oct 2016): MyFitnessPal; Fitbit; Lose It!; MyPlate; Lifesum	Food diary app	IOS	Nutrition Data System for Research (NDSR)	*Construct validity:* Correlations between methods ranged from 0·73 to 0·96 for energy and macronutrients, across the apps. For each app, at least one nutrient intake calculation was found to be significantly lower than the calculations from the Nutrition Data System for Research (NDSR). They were: total protein, total fat, Na, and cholesterol for MyFitnessPal; dietary fibre for Fitbit; total protein, total fat, Na, sugars, cholesterol and saturated fat for Lose It!; Na and dietary fibre for MyPlate; and total fat for Lifesum. *With respect to the extent to which foods and food amounts entered into each app matched those in the NDSR foods reports, Most foods and food amounts (78-83%) entered into each app closely matched those in the Nutrition Data System for Research. MyFitnessPal had the highest*

													percentage of close matches (83 %). Across apps 13-17% of entries poorly matched NDSR food amounts and 4-6% poorly matched food descriptions.
Bucher	2017	Switzerland	50	60.33%	NR	NR	18 - 60		e-CA	Food diary app	NR	paper-based food record and 24 hr phone diet recall	*Measurement error:* Bland-Altamn plots found good agreement for energy, macronutrient, and food group intake assessed by the app versus the 24-hour recall interviews. *Construct validity:* No significant differences between energy, nutrients and foods groups were found between intakes assessed via the app vs the 24-hour recall interviews, with the exception of lipid intake were the app yielded a significantly lower result. *Criterion Validity:* Compared to the real weight of food items participants overestimated portion sizes by 3% in the app and by 3.9-4.7% using paper-based records. The foods and drinks displayed were equivalent to 2372 kcal. mean estimations of energy using the app was 2090-2150 kcal and 2043-2300 kcal using paper-based food records.
Bruening	2016	USA	109	67.00%	18.83	0.61 (diet) and 0.50 (PA)	NR	University students	devilSPARC	ecological momentary assessment app	Android or IOS	Online version of the Automated Self-Administered 24-hour (ASA24) dietary recall system	*Construct validity:* Dietary intake data reported through the app reflected eating choices also captured by the 24-hour recall, with an average of 86.6% of foods reported in both systems. This ranged from 79% for entrées and 94% for fruit and vegetables.
Carter	2013	United Kingdom	50	72.00%	35	9	NR	University staff and students	MMM (My Meal Mate)	food diary app	Android	24 hr food recall (phone call)	*Measurement error:* Bland–Altman analysis showed wide limits of agreement between the methods, but showed that bias between the methods appeared consistent over different levels of energy intake. Construct validity: The correlations between methods energy and macronutrients were moderate to high (r 0·63–0·83). Mean totals for energy and macronutrients were not significantly different on day 1 of the study, however mean daily energy and fat intakes were found

													to have significantly different on day 2 of the study.
Bejar	2017	Spain	119	71.40%	21.9	3.2	NR	University students	e-EPIDEMIOLOGY	food diary app	Android	Food Frequency Questionnaire (FFQ) poorly described - the paper-based FFQ used is very short<br><br>and simple (containing only twelve items)	*Construct validity:* Moderate correlations between methods was found for all food and drink groups assessed (mean r = 0.73). Cross-classification analysis showed good agreement between the two methods (8.2% of the participants were incorrectly classified into 2-4 categories apart). The average weighted kappa statistic showed moderate agreement between methods (k = 0.60), with good agreement for fruit, vegetables, fish, soft drinks and alcoholic beverages (k = 0.61-0.68) and moderate agreement for legumes, chicken/turkey, red meat, sweets and prepared foods (k = 0.52-0.59).
Bejar	2017	Spain	187	64.20%	28.2	10.9	NR	University staff and students	e-12HR	food diary app	Android	Food Frequency Questionnaire (FFQ) poorly described - the paper-based FFQ used is very short<br><br>and simple (containing only twelve items)	*Construct validity:* Moderate to high correlations between methods were found for all food groups assessed (mean r = 0.70). Cross-classification analysis showed good agreement between methods (9.8% of participants misclassified). The average weighted kappa statistic showed moderate agreement between methods for all food groups (k = 0·55).
Ambrosini	2018	Australia	50	82.00%	31	NR	NR		Easy Diet Diary	food diary app	iOS	To provide an estimate of usual dietary intake for comparison with the RFD 4-d diary, two 24-h dietary recalls<br><br>were collected	*Measurement error:* Bland-Altman plots showed there was acceptable group level agreement between methods with little evidence of systematic bias between methods, with the exception of added sugars, where individual differences between methods increased significantly as intakes of added sugars increased. *Construct validity: Moderate* correlations between

													methods were found for all food groups , from r = 0.42 for iron density, to r = 0.79 for protein (mean r= 0.61). The average difference between methods in total energy intake (268 kJ/d) was deemed to be acceptable.
Ali	2017	UK	54		57		45.3–68.7	Family history, established genetic risk, minor macular abnormalities or established macular degeneration.	NR	food diary app	Android or iOS	Pen and paper records of food intake	*Measurement error:* Bland-Altman plots demonstrated clinically acceptable agreement for all micronutrients, more than 90% of all values were within the limits of agreement. Reasons for discrepancies included: when using the app. patients were more specific with the type of food, were more likely to enter a weight for the food, logged their snacks and drinks more reliably, and were more likely to log constituents ingredients of a meal, such as dressings and type of oil used.
Lancaster	2019	Australia	48	68.75%	64	5.5	55-75		Research Food Diary (RFD) app	food diary app		Dietitian-assisted reported food record (participants own food records updated after participant interview with research dietitian e.g. to identify any omitted food items, clarify volumes etc)	Measurement error: 83% of participants submitted food diaries via the app with at least one error. While the mean energy intake of the self-reported food diaries via the smartphone was non-significantly lower, compared to the adjusted or dietitian-assisted food diaries, ten micronutrients, serves of grains and cereals, meats and alternatives, dairy foods and added sugars were significantly lower in self-reported diaries compared to the adjusted and dietitian-assisted diaries. Records from day 1 were found to contain more errors (n = 45), compared to the second (n = 29) and third days diary (n = 32).

Wellard-Cole	2019	Australia	189	54.00%	NR	NR	18-30	Young adults	Eat and Track Smartphone Application (EaT App)	food diary app	Android or iOS	N	*Measurement error*: Bland–Altman plots showed wide limits of agreement but no obvious biases for nutrient densities except carbohydrate in males. Males had a higher mean difference than females. *Construct validity:* Correlations between methods were moderate to strong, ranging from 0.56 for total fat to 0.82 sugars. A moderate correlation between methods was found for total energy, r= 0.67. Significantly more energy was recorded using the 24-h recalls than the EaT app for the total sample ($p < 0.001$), females ($p < 0.01$) and males ($p < 0.001$)
Liu	2019	Taiwan	105	66.70%	35	Over all SD 19.5	18-29 AND 55-73	University students	NR	food diary app	Android	Actual food served (consistent across all Ps)	*Measurement error & criterion validity:* Participants using the self-chosen tab app and the autonomous exhaustive list app were able to label food items with 97.77% (1228/1256) and 98.53% (1214/1232) accuracy respectively.
Lemacks	2019	USA	15	78.60%	26.2	NR	19-45		Bridge2U	food diary app	NR	Actual food consumed (as measured using pre-portioned food provided in lab setting and ratings of food waste my research dietitians) & 24 hour recall	*Measurement error:* Bland Altman plots showed wide limits of agreement between methods, which were not statistically significant but may have practical limitations for individual dietary assessment. *Criterion validity: Moderate correlations between mean energy, carbohydrate, and protein intakes (r = 0.57–0.60, p < 0.05)* as measured by the mfood log compared to actual energy and nutrient values of the control meal. Total energy as estimated by mfood log was significantly lower than the control meal.
Fallaize	2019	UK	20	75.00%	36.3	NR	NR		Samsung Health (S Health; Samsung), MyFitnessPal (MyFitnessPal, Inc), FatSecret (Secret Industries Pty Ltd), Noom Coach (Noom	food diary app	NR	research standard for dietary analysis of WFRs (Dietplan6	Measurement error: Bland-Altman plots found good levels agreement between the reference method (weighed food records) and S Health, FatSecret, and Noom Coach for estimates of energy. For MyFitnessPal and Lose It! 10% of cases were found to fall outside of the limits of agreement, indicating less agreement between methods.

									Inc), and Lose It! (FitNow, Inc),				Bland-Altman analysis revealed potential proportional bias for vitamin A. Construct Validity: For estimations of energy intake there were strong correlations (r=.79 to r=.91) between methods for the apps S Health, MyFitnessPal, FatSecret, and Noom Coach. No significant differences in estimation of energy and saturated fat intake between the weighed food records and all the diet apps were found. However, estimates of protein and sodium intake were found to be significantly lower using Lose It! and FatSecret. Estimates for carbohydrate, fat, fibre, and sodium were also significantly lower using Lose It! Estimates of calcium, iron, and vitamin C were significantly lower using Samsung Health and MyFitnessPal.
Hezarjaribi	2019	USA	13	38.50%	NR	NR	18-35	University students	EZNutriPal	food diary app - unstructured data entry	Android	Participant labels of entered text. Know food items to be entered.	*Construct validity:* The final model of EZNutriPal accurately identified 89.7% of food items described by participants entering data into the app using free speech or text on average. *Criterion validity:* The Speech2Health app identified 3.4× more than the actual number of food items contained in test sentences. EZNutriPal identified 0.8× less than the actual number of food items contained in test sentences.
Chen	2019	Australia	47	80.85%	NR	NR	18+		MyFitnessPal	food diary app	Android or iOS	24 hour recall test (The National Cancer Institute Automated Self-Administered (ASA24)-Australia)	Measurement error: Bland-Altman plots showed no proportional bias for energy or any of the nutrients assessed, however, wide limits of agreement were observed. Construct validity: Correlations between methods were weak (r = 0.21–0.42) for energy and macronutrients assessed. Significantly lower values or energy and all macronutrients were recorded via MyFitnessPal compared to 24-h recalls.

Swendeman	2018	USA	42	100.00 %	31.2	NR	20-43	Women with a child below 18 years who is living at home	Ohmage	ecological momentary assessment app	Android	Anthropedic measures (weight, BMI, blood pressure) blood spot biomarkers & food frequency questionnaires from the California Health Interview Survey.	Construct validity: Moderate correlations between ecological momentary assessment of diet quality and dietary recall measures was found (0.34 for Fruit and Vegetables, -0.52 for foods with high sugar content and -0.42 for fast food). Criterion validity: Weak correlations between diet quality recorded via the app and diet related anthropedic measures were found (r=−.32 for systolic blood pressure and r=−.34 for C-reactive protein level).
Rodder	2018	USA	172		NR	NR	NR	physician assistant (PA) and clinical nutrition (CN) students	MyNetDiary	Food diary app	NR	Gold standard nutritional estimation tool - SuperTraker/NutriBase software	Construct validity: Moderate to strong correlations between methods was found (mean r = 0.67). Strong positive correlations between methods were found for sodium, calories, carbohydrates, protein, Vitamin C and fats (r =0.73 to r = 0.88), Moderate positive correlations were found for fibre, potassium, Vitamin A , Vitamin D, irons and magnesium (r= 0.45 to r=0.65).
Bejar	2019	Spain	203	56.70%	32	11.4	NR	University staff and students	e-12HR	Food diary app	Android	Validated dietary record tool (on paper)	Construct validity: For all food groups and all participants, the mean correlations between the app and food frequency questionnaire (FFQ) was 0.67. Cross-classification found on average, 50.7% of participants were classified into the same category and 90.2% within the nearest category. Mean weighted kappa was 0.49 indicating good agreement between methods. For the app versus 4 days dietary records, mean correlation was 0.65 . On average, 50.0% of participants were classified into the same category and 88.2% within the nearest category. Mean weighted kappa was 0.50, indicating good agreement between methods.

Chmurzynska	2018	Poland	62	42.00%	25.8	5.4	20-40		NR	Food dairy app - for high fat foods	NR	the Block Screening Questionnaire for Fat Intake (BSQF),	*Measurement error:* Bland-Altman plots showed the mean differences between methods was similar for normal weight and overweight and obese participants, however the agreement limits were wider in the overweight/obese group than in the normal weight group. *Convergent validity:* Moderate correlations between methods was found for all participants (0.38 to 0.42). When stratified by BMI, the correlation between methods for overweight or obese participants was no longer significant. Correlations between methods for normal weight participants was 0.55 to 0.67.
Hezarjaribi	2018	USA	30		NR	NR	NR	NR	Speech2Health	Food diary app - unstructured data entry	Android	Lab studies: known phrases describing foods used by participants. Field study: Ground-truth labelling that allowed users to correct any errors in food intake calculated from voice data.	Construct validity: App achieved an accuracy of 92.2% in computing calorie intake.
Bejar	2018	Spain	87	64.00%	19.2	3.3	NR	University students	e-12HR	Food diary app	Android	Validated dietary record tool (on paper)	Construct validity: The average correlation for between methods all foods and drinks was strong for the app versus the food frequency questionnaire (FFQ) (r= 0.70) and moderate for the app vs the four dietary records (r=0.63). Cross-classification analysis showed on average 51.5% of participants were classified into the same category, 91.8% within the nearest category and none in the extreme disagreement category. The average weighted kappa was 0.47 (app vs dietary records) to 0.51 (FFQ), indicating good agreement between methods. For e-12HR versus the four dietary records, for all food and drink groups, the average SCC was 0.63. Cross-classification analysis revealed that the average percentage of individuals classified

													in the exact agreement category was 47.1%; exact agreement + adjacent was 89.2%; and no participant (0%) was classified in the extreme disagreement category. The average weighted kappa was 0.47."
Ahmed	2017	Canada	18	22.00%	34	11	NR	Canadian Armed Forces Personnel	MyFitnessPal	Food diary app	Android	Gold standard measured food intake/waste method (wFR - weighed food record)	Measurement error: Bland-Altman plots for energy, macronutrient, and micronutrient intakes found data for most participants were within the limits of agreement with few outliers. There was no evidence of proportional bias. Criterion validity: Correlations between methods were very strong h for both macro- and micro-nutrients (0.963 to 0.999). The differences between the methods were not significantly different (p > 0.05) for nutrients energy, carbohydrates, fat, saturated fat, protein, vitamin A, vitamin C, calcium, iron, and sodium. For all of these nutrients, the app yielded lower intakes than the measured food intake/waste method. The mean difference between methods for overall energy intake was not significant.

Diet - Active Objective

Zhang	2015	United States	0	NA	NA	NA	NA	NA	Snap-n-Eat	Automatically analysed food photography: User captures image of food via the app and smartphone camera. The app then detects salient regions and subtracts the background accordingly. The image is segmented into regions from which features are extracted and used to classify these regions into different kinds of foods. The system then determines portion size which is used to estimate the calorific and nutrition content of the food .	Android	known food items presented in images within lab-based experiments	*Criterion validity:* App achieved 85% accuracy when detecting 15 different kinds of foods from images
Rhyner	2016	Switzerland	19		40.5	11.5	NR	Diabetics	GoCARB	Automatically analysed food photography: Reference card placed next to the dish and two images taken using the mobile phone. A series of computer vision modules detect the plate and automatically segment and recognize the different food items, while their 3D shape is reconstructed. Finally, the carbohydrate content is calculated by combining the volume of each food item with the nutritional information provided by the USDA Nutrient Database for Standard Reference.	Android	Construct validity - GoCARB compared to participants estimating carbohydrate content themselves as well as compared to the ground truth (measured by weighing the meals and calculating the carbohydrates using the USDA nutrient database.	Measurement error: The mean absolute error for the estimation of grams of carbohydrates within the app was significantly less than participants' own estimations. The corresponding mean relative error in carbohydrate estimation was 54.8% (SD 72.3%) for the participants and 26.2% (SD18.7%) for GoCARB. The distribution of errors for participants' own estimations was broad with outliers up to 200 grams of carbohydrate. For the app errors were symmetric and concentrated around zero.

Nicklas	2017	United States	39	44.00%	5.4	0.6	3 - 5 year olds		NA	Manually analysed food photography: Caregivers captured before and after pictures of their child's food intake using an iPhone. Photos were wirelessly transmitted to trained raters who estimated portion size and energy and macronutrients were calculated.	NR	Doubly Labelled Water to estimate energy intake	Measurement error: Bland-Altman plots for energy intake showed that there was a slight positive trend of the differences between methods as the mean energy intake of the two methods increased from 1000 to 1800 kcal/d, but this bias was not statistically significant. Criterion Validity: The Remote Food Photography Method was found to significantly underestimate mean daily energy intake compared to energy intake calculated via the doubly labelled water method by -15.6% (group mean error) ($p < 0.0001$).
Liu	2016	Taiwan	108	69.00%	21.3	NR	NR	University students	NR	Automatically analysed food photography: Two new methods for dietary food measurement examined: An Interactive Photo Interface IIPI) method and a Sketching-based interface (SBI) method. The IPI presented users with images of pre-determined portion sizes of a specific food and allowed users to scan and then select the most representative image matching the food that they were measuring. The SBI required users to relate the food shape to a readily available comparator (e.g., credit card) and scribble to shade in the appropriate area.	NR	Known weight of food items, traditional life size photo method (where an album demonstrating different food portions, commonly used by dietitians to help people select the type of portion they are eating, is used).	Construct validity: The overall accuracies of the IPI, SBI, and traditional life size photo method were 66.98%, 46.05% and 72.06% respectively. The SBI method was significantly less accurate than traditional life size photo method ($p<.0001$). There was no significant difference in the overall accuracy of the IPI and traditional life size photo method and no significant differences for each shape or cutlery category with the exception of the pork strip in the irregular strip category ($p < 0.05$).

Martin	2009	USA	52		32.4	1.5	18-54		NA	Manually analysed food photography: Participants received 4 - 6 automated prompts to take photographs of their meals and send them to researchers. Participants provided with a pen to standardize the distance of the camera from the food and instructed to take photographs at a 45 degree angle. Photographs were rated by 3 research dietitians to estimate the energy intake.	NR	Known energy intake of foods calculated by weighing food provided and food waste.	Reliability: Energy intake estimated with the RFPM was reliable over the 3 days of testing. Reliability in lab conditions was .62 and .68 for the free living condition. Criterion validity: Energy intake estimated with the RFPM correlated highly with weighed energy intake in laboratory conditions, though the RFPM significantly underestimated energy intake. In free living conditions energy intake also significantly underestimated energy intake. Analysis indicated that there was no significant associated between body weight, or age and the RFPM's error. In the dine in group, error was significantly larger for women.
Martin	2012	USA	90	82.75%	42	study 1: 14.3, study 2: 12.8	18-65	Overweight and obese adults	NA	Manually analysed food photography: Prompts automatically sent to participants to remind them to capture images of their foods and to send these images to the research staff. Participants' energy and nutrient intake then estimated by comparing their food images to images of foods with a known portion size.	NR	Doubly Labelled Water to estimate energy intake, directly weighing food and food waste.	*Reliability*: Over 6 days, daily energy intake estimated with the RFPM was reliable, with an intraclass correlation coefficient of 0.74. *Measurement error:* The error associated with the RFPM was found to be consistent over levels of energy and nutrient intake. *Criterion validity:* When standard prompts were used RFPM underestimated energy intake. When customized prompts were utilized, energy intake estimates did not differ significantly between methods. In a free living context while the RFPM underestimated energy intakes, this difference was not significant. Similarly, in a lab-setting energy intakes estimated by the RPFM did not differ significantly to estimates calculated by directly weighing foods. With the exception of vitamin A and cholesterol, no significant differences were detected between estimates of macro and micronutrients with the RFPM compared to directly weighing foods.

Kong	2017	China	117	59.00%	21.4	2.5	NR		NA	Manually analysed food photography: Photographs of meals sent to researchers via WeChat	NR	Weighed food record	*Measurement error:* The Bland Altman plots showed good agreement between the two methods and also indicated that the differences (including the outliers) were random and did not exhibit any systematic bias. *Criterion validity: Very strong correlations between methods were found* for energy, protein, fat and carbohydrate content (0.997, 0.936, 0.996, and 0.999, respectively).
Huang	2015	Australia	0		NA	NA	NA	NA	NR	Automatically analysed food photography: User takes a photo of food with their smartphone. The photo is then automatically analysed in the app through three steps: food classification, volume estimation, and carbohydrate calculation. According to the food type, the application searches a food database to obtain the density of the food and carbohydrate concentration. Finally, the amount of carbohydrates contained in the food is calculated, and reported to the patient.	Android	Known energy and nutrient content of food calculated via direct weighing of foods	Criterion validity: The app estimated the volume of foods with an average error rate of 6.8% (range 4.3% to 13.9%), and estimated of carbohydrate content with an average error rate of 8.18% (range 16.1% to 2.45%). The accuracy of the app in identifying food items varied. Ranging from 70% accuracy in classifying tomatoes and 100% accuracy in classifying avocados, pineapples, and kiwifruits. The overall accuracy was 90%.
Delisle Nystrom	2016	Sweden	39	44.00%	5.5	0.5	NR		NA	Manually analysed food photography: Photos of children's food taken by parents and sent to researchers for analysis.	NR	24hr telephone dietary recalls, doubly labelled water.	Measurement error: Bland Altman plots for energy intake found wide limits of agreement but no evidence of systematic bias. Construct validity: Moderate to strong correlations between the analysed food images and 24 hour recalls were found for all eight food categories (r=0.665 to r=0.896). Criterion validity: Mean energy intakes estimated by the analysed food images were not significantly different to total energy

													expenditure as calculated via the doubly labelled water method.
Boushey	2017	USA	45	66.00%	33	12	21 - 65		my Food Record (mFR)	Manually analysed food photography: Participants use the app to capture before and after images of all eating occasion. Participants were provided with a fiducial marker to use in each of the photos to assist with analyses of portion sizes. Three trained analysts estimated energy and nutrient content from the images sent to a secure server via the app.	NR	doubly labelled water method to estimate energy expenditure.	Measurement error: Bland–Altman plots did not show evidence of systematic bias, reporting accuracy was consistent over all energy intake levels. Criterion validity: A moderate statistically significant correlation between methods was found (r 0.58). Mean values for energy intake calculated via the app was found to be significantly less than total energy expenditure calculated via doubly labelled water. Difference between methods was found to be greater for men than women.
Costello	2017	United Kingdom	12	0.00%	17.9	0.5	16-18	Elite adolescent male athletes	Snap-N-Send	Manually analysed food photography:	NR	weighed food records	Measurement error: Bland-Altman plots assessing agreement methods found evidence of small, standardised bias. Construct validity: Correlations for energy intake between the app and weighed food records were very strong, ranging from r=0.93 to r=0.98.

Ashman	2017	Australia	25	100.00 %	NR	NR	20.4 - 50.4	Pregnant women	Evernote	Manually analysed food photography: The Evernote app was used to record all eating and drinking occasions. Users took a phone image of the consumed items placed next to a reference object of known dimensions. Text or voice descriptions added to the image to support the identification of items in the image. Users also instructed to record images of all food and drink leftover, and any second servings. Images then assessed by dieticians and energy and nutrient intakes calculated using the FoodWorks® nutrient composition software.	NR	Three 24-hr recalls - with energy and nutrient intake calculated using the FoodWorks software	*Measurement error:* Bland-Altman plots for energy and macronutrient intakes indicates the majority of values were within the acceptable limits of agreement. *Construct validity:* Moderate to strong correlations between methods were found for energy, macronutrients and fibre (r = 0.58–0.84), and for micronutrients both with supplement use included (r = 0.47–0.94) and without supplement use included (r = 0.40–0.85). No significant mean differences in nutrients between the two methods were found, with the exception of total fat and saturated fat. However, mean differences were small and not clinically important for any nutrient.
Zhu	2010	USA	78	66.00%	NR	NR	18+	NR	NR	Automatically analysed food photography: Images of meals captured using the smartphone camera. Each food item is in the image is then segmented, identified, and its volume is estimated. “Before” meal and “after” meal images used to estimate the food intake. From this information, the energy and nutrients consumed are determined.	iOS	Known food items and their nutrient information in images were from previous lab studies	Criterion validity: The app accurately identified between 84.2% of foods (with 10% training set) and 95.8% (with 50% training set). Mean percentage error of volume estimates was 5.65%, ranging from 0.86% for estimating volume of orange to 14.6% for plums. For estimating mass the system had a % error of 3-56%. With 10% training data, the app reported within 10% margin of the correct nutrient information, with 25% raining data, the app improved to within 3% of correct nutrient information and within 1% with 50% training data.
Rollo	2011	Australia	10		NR	NR	59-70	Type 2 Diabetes	Nutricam	Manually analysed food photography: App allows users to capture images of food items before consumption, store a voice recording explaining the contents. Images are then sent to	NR	Food diary (based on objective measurement of food using household utensils and food analysis program [FoodWorks version 5.1, Xyris Software, Brisbane, Australia])	*Construct validity:* Compared to the food diary, energy intake was under-recorded by 649 kJ (SD 810) using the mobile phone method. However, there was no trend in the difference between dietary assessment methods at levels of low or high energy intake.

										a website for analysis by a dietitian.			
Prinz	2019	Germany	66	63.64%	22	NR	NR		NA	Manually analysed food photography: Users took photos with their smartphone's built-in camera. Each picture had to be taken at an angle of approximately 45°. To better estimate serving sizes, a reference object (2 € coin) had to be placed in the upper corner of each picture. At the end of the day, participants submitted their images via email to the researchers. Images were evaluated to determine if all meal components could be identified and participants followed up to clarify if they could not. Pictures were analysed by a nutrition scientist and nutrient content calculated using the computer program DGExpert (version 1.6.7.1) developed by the German Nutrition Society.	NR	Weighed food records	*Measurement error:* Bland-Altman plots for daily energy and macronutrient intakes indicated good agreement between methods, however increasing intake level, underestimation by the app was present. *Criterion validity:* Very strong correlations between methods were observed for energy (r=0·991), carbohydrate (r=0·980), fat (r=0·972), protein (r=0·988), fibre (r=0·941).
Rollo	2015	Australia	10	40.00%	61.2	6.9	48-69	People with Type 2 diabetes.	Nutricam Dietary Assessment Method (NuDAM)	Manually analysed food photography: App allowed users to capture a photograph of food items before consumption and store a voice recording to explain the contents of the photograph. This information was then	NR	Doubly Labelled Water & Weighed food records	*Criterion validity*: Correlations between the NuDAM and weighed food records were mostly moderate for energy (r = 0.57), carbohydrate (r= 0.63), protein (g/day) (r = 0.78) and alcohol (r = 0.85), with a weaker relationship for fat (r = 0.24). Overall mean energy intake calculated by the app and weighed food records were

										sent to a website where it was analysed by a dietitian			both significantly lower than total energy expenditure calculated via the doubly labelled water method. Participants who were found to under-report using the app also under-reported via the weighed food records.
Kato	2016	Japan	8		NA	NA	NA		DialBetics	Manually analysed food photography: Smartphone app facilitates photos of meals to be taken. Three registered dietitians evaluated each dish from those photos, naming the dish and estimating it's nutrient content.	All Smartphones	A Weighed Food Record	*Measurement error*: Bland-Altman plots showed good agreement between the two methods. They indicate that the differences were random and did not demonstrate any systematic bias. *Criterion validity:* Strong correlations between methods were found for energy (r=0.92) and macronutrients r=0.84 for fat and r=0.93 for carbohydrates. The app recorded higher macronutrient values than the weighed food record, except for energy and protein.
Most	2018	USA	23	100.00 %	28.3	1.1	18-40	Pregnant obese women	SmartIntake	Manually analysed food photography: Participants were instructed to take images of all eating occasions as well as any food waste. A reference card was placed next to the food items photographed at each eating occasion, which allowed for accurate portion-size estimation. Images were automatically transmitted via cellular networks or Wi-Fi from the application to the Researchers and reviewed at the conclusion of the assessment period by the participant and study staff. Food items were categorized as meals (breakfast, lunch, or dinner) or as snacks. Energy intake	iOS	total daily energy expenditure measured via doubly labelled water	Criterion validity: Compared with total energy expenditure measure via doubly labelled water the app captured only 63.4% of energy intake and was not equivalent to total energy expenditure within a 20% equivalence bound. There was a significant, systematic reporting bias across the observed range of energy intake that was positively correlated to pre-pregnancy BMI. African-American women reported significantly lower energy intake than did white women and Asian and biracial women.

										was estimated by trained and certified raters.			
Schiel	2012	Germany	124	56.00%	13.5	2.8	NR	Children and adolescents with overweight and obesity	DiaTrace	Manually analysed food photography: Children and adolescents took photos at the beginning of each meal. The photos were analysed separately and anonymously by two educators specialized in nutrition to calculate their energy intake.	NR	Unspecified self-report measures of diet	Construct validity: There was a non-significant difference between methods for energy intake. On average patients' estimations were slightly lower than then those via the app.
Schiel	2010	Germany	30	46.70%	14	3	NR	Children and adolescents with overweight and obesity,	DiaTrace	Manually analysed food photography: Children and adolescents took photos at the beginning of each meal. The photos were analysed separately and anonymously by two educators specialized in nutrition to calculate their energy intake.	NR	Self-assessed caloric intake. Patients were taught to estimate the calorie content of their meals based on the recommendations of the nutrition experts.	Construct validity: There was no significant difference between methods. Participants estimated their caloric intake to be lower than the calculated by the app.

Alcohol - Self-Report

Wray	2018	USA	15	0.00%	24.2	3.8	18-31	Males reporting at least one condomless anal sex event with a casual male partner in the last 30 days, and "at-risk" drinking.	Metricwire	Smartphone app used for survey research	NR	Timeline follow back-like approach for the previous 30 days	*Construct validity: Moderate to strong correlation between methods found* (r = 0.55–0.88). However results provided evidence of underreporting on the Timeline Follow Back.
Paolillo	2018	USA	35	26.00%	57.5	6.1	50-74	22 HIV-positive, 13 HIV-negative	NR	Ecological momentary assessments (EMAs): Prompted to answer EMA surveys four times per day over 14 days. Schedule of EMA surveys was customized to each participant's sleep-wake schedules.	Android	NA	Construct validity: The proportion of ecological momentary assessment surveys on which participants endorsed using alcohol was moderately correlated to reported number of drinking days (r= 0.52) and total drinks consumed(r= 0.42) on the Timeline Follow Back.
Monk	2015	UK	69	41.00%	21.47	4.47	18-36	University students	NR	Alcohol use diary app: Participants prompted hourly to report alcohol use, location and surroundings.	No specific system - 'Smartphones' in general	24hour and 1 week recall conducted via email	Construct validity: Moderate correlations between methods found (r=0.52), however results also suggested participants under-report the number of drinks they consume via both daily and weekly retrospective reports compared to real-time reporting in the app.
Dulin	2017	USA	25	48.00%	33.6	6.5	22 - 45	Heavy drinkers	LBMI-A (Location-Based Monitoring and Intervention System for Alcohol Use Disorders)	Intervention & alcohol use diary app: App provides access to seven treatment modules to enhance motivation to change, improve social support for sobriety, develop awareness of alcohol triggers, and improve coping methods. Each day at a pre-set time, participants receive a prompt via the app to record number of standard drinks consumed during the previous 24 hours.	NR	Timeline Follow Back	Measurement error: Correlations between methods was found to decrease significantly as more time elapsed between consumption and administration of the timeline follow back (B [SE] = .34 [.17], p = .040). Construct validity: Moderate to strong correlations between methods were found for drinks per drinking day (r= 0.49- 0.74), percentage Heavy drinking days (r = 0.74 - 0.87); and percentage days abstinent (r =0.76 - 0.92).

Poulton	2018	Australia	671	70.00%	23.12	7.24	16-56		CNLab-A app	Alcohol use diary app	iOS or Android	Timeline Follow Back, Alcohol Use Questionnaire.	*Construct validity:* Significantly greater percentage of drinking days reported via the app than the Timeline Follow Back (26.44% vs 24.79%). Significantly higher total intake reported via the app than via the Timeline Follow Back (24.26 vs 20.30 standard drinks). A There was no difference in average weekly consumption recorded via the app or the Alcohol use Questionnaire. Avery hourly rate of alcohol consumption was significantly higher when measured via the app as compared to the Alcohol Use Questionnaire.
Luczak	2018	USA	1		NR	NR	NR	a single expert subject - one of the authors of the study	Intellidrink	Alcohol use diary app & breath alcohol concentration calculator: App requires user to enter demographic information and data for each drinking episode.	iOS	Previously validated Breath alcohol estimator software developed by authors	Construct validity: The app produced similar estimated breath alcohol concentration curves to the comparison measure. The app captured peak eBrAC to within 0.0003%, time of peak eBrAC to within 18 min, and area under the eBrAC curve to within 0.025% alcohol–hours as calculated by the breath alcohol concentration estimator software.
Kizakevich	2018	USA	72	14.00%	NR	NR	18-66	Military personnel, 91% had been previously deployed.	PHIT for Duty	Intervention app: App delivers mindfulness-based relaxation, behavioural education in sleep quality and alcohol use, and surveys regarding a range of physical and mental health factors, including administering the Alcohol Use Disorders Identification Test (AUDIT) to assess alcohol use.	iOS and Android	AUDIT and AUDIT-C completed using pen and paper	Construct validity: Strong correlation between AUDIT scores completed via the app and pen and paper (r=0.97). No significant differences between methods for alcohol use.
Barrio	2017	Spain	24	50.00%	48	11.3	NR	people with Alcohol Dependence	SIDEAL (Support to the Alcohol Dependent Patient)	Alcohol use diary app: With icons representative of alcoholic drinks, the patient records his/her alcohol consumption. The system automatically converts the information into standard units and	NR	Timeline follow-back administered at the end of the study	Construct validity: Strong correlations between methods for alcohol consumption found (r=0.95)

										graphically displays this information, where an agreed upon limit is also displayed.			
Bernhardt	2009	US	168	50.30%	19.96	1.35	NR	College students	HAND - Handheld Assisted Network Diary	App delivers surveys regarding alcohol consumption the previous day	NR	Timeline Follow back and Daily Social Diary	Construct validity: Mean total drinks did not differ significantly between methods.

Alcohol – Active Objective

Matsumura	2009	Japan	11	0.00%	23.3	3.6	NR	No current or past history of drug or alcohol abuse	Spiral for iPhone	A psychomotor performance test, measuring accuracy and reaction time	iOS	Known blood alcohol concentrations of participants	Reliability: Predicted reliability (ρ) for Spirals for the durations of being on track, out of track and number of deviations were .49, .83, and .86, respectively. Responsiveness: For blood alcohol concentrations close to 0.1% (mean =0.111%) participants' performance on all tests, including the Spiral for iPhone was significantly worse than at blood alcohol concentrations of 0.0%. For blood alcohol concentrations clos to 0.06% (mean = 0.062%), performance on all tests, except Spiral for iPhone, was significantly worse than performance at blood alcohol concentrations of 0.0%.
Kim	2017	USA	0		NA	NA	NA	NA	SPAQ2	Custom built smartphone attachment and smartphone app to accurately capture an image of saliva alcohol concentration test strips and identify the relevant alcohol concentration.	Android	Known saliva-alcohol concentrations	Reliability: Between 93.3 - 100% accuracy achieved across phone types for one data set. Criterion validity and responsiveness: Average classification rates of 100% accuracy were achieved for standard saliva-alcohol concentrations (0.0%, 0.02%, 0.04%, 0.08%, 0.30%). Average classification rates of 80% accuracy were achieved for intermediate saliva-alcohol concentrations requiring finer discrimination (0.01%, 0.03%, 0.06%, and 0.15%).

Alcohol – Passive Objective

Arnold	2015	USA	6	NR	NR	NR	6 'users' - this study was measuring the app constantly over the course of 2 weeks rather than looking at individuals	AlcoGait	App runs in the background of user's phones. Accelerometer and gyroscope data collected and information about user's gait generated. In-app surveys collect information about user's alcohol use.	Android (not specified)	AUDIT and AUDIT-C completed using pen and paper & saliva alcohol concentration measurements	Criterion validity: Without training data app had an accuracy of 56.0% (F-score = 0.629). After training on 30-data points app had an accuracy of 70.0% (F-score - 0.786). Responsiveness: After training, app was shown to have good ability to discriminate between different levels of impairment (AROC = 0.825).
Santani	2018	Switzerland	241	Nr	Nr	16-25	young people from major cities in Switzerland	Sensor logger & Drink logger	Sensor Logger: App runs in the background of user's smartphones collecting information from various sensors (GPS, Wi-Fi, accelerometer, Bluetooth, battery, screen, app usage) during the weekend nights from 8 PM until 4 AM. Drink logger: App allows users to respond to various in-situ surveys including reporting their drink consumption.	android	Self-reported alcohol use - collected via the Drink Logger app.	*Construct validity:* Accelerometer data was the most informative feature, with 75.8 percent accuracy in classifying drinking episodes. This was followed by location, Wi-Fi and Bluetooth logs with 68.5%, 65.2% and 64.2% accuracy, respectively.

Bae	2018	USA	30	50.00%	23.1	1.7	NR	ED patients and college students	AWARE	App runs in the background of users' phones and automatically records data from participant's phone sensors including: day of week, time of day, accelerometer, gyroscope, calls, texts, and keystroke speed.	iOS & Android	Daily ecological momentary assessments: Brief survey administered via text message each morning asking participants to specify if they had drunk alcohol in the previous 24 hours and approximately what time they started and finished drinking and how many drinks they had in that period. Allowed researchers to classify each 30 min lot in the study as non-drinking, high or low drinking episodes	Construct validity: Time of day and day of the week were the most informative features, with 90% accuracy in classifying drinking episodes. Self-reported alcohol use (non-drinking, low-risk drinking, high-risk drinking) weakly, but significantly, correlated with time of day (r=0.11) and day of week (r=0.06), screen interaction (r=0.07) and transitions between walking and in vehicle (0.03). The best performing model to detect drinking overall used 3 days of historical data and correctly classified 30 minutes windows of time as non-drinking with 98.5% accuracy, low-drinking with 70.2% accuracy, high-risk drinking with 84.4% accuracy (kappa=0.804, ROC=0.961).
McAfee	2017	USA	33	39.40%	20	1.32	18-22	Psychology students	AlcoGait & AlcoWear smartwatch app	AlcoGait: App runs in the background of user's phones. Accelerometer and gyroscope data collected and information about user's gait generated. In-app surveys collect information about user's alcohol use. AlcoWear: Collects accelerometer and gyroscope data from users' smartwatches, in addition to the information collected from users' smartphones via the AlcoGait app. Users this information to infer users' blood alcohol concentration levels.	Android	Known level of impairment - induced using Sensor-impairment goggles that simulates the effects of alcohol consumption of the body, corresponding to different levels of intoxication / blood alcohol concentrations	Construct validity: AlcoGait app found to infer participant's blood alcohol concentration ranges as 0.00-0.08%, 0.08-0.15%, 0.15-0.25% or 0.25%+ with 89.45% accuracy. AlcoWear smartwatch found to infer participant's blood alcohol concentrations as above or below 0.08% with 79.8% accuracy.

Tobacco - Self-report

Swendeman	2015	USA	34	33.00%	NR	NR	23-64	HIV positive, current alcohol or substance use	Ohmage	App-based survey platform	NR	2 week web-based recall	*Measurement error:* Discrepancies between daily, app based reports and 2 week- web based recall increased as the number of missing daily reports increased, which suggests that participants tended to report on days they used substances compared to days they did not use. *Construct validity: Correlations between methods was high for tobacco (r=0.92) and moderate for alcohol (r=0.64). Significantly fewer mean days of tobacco use were reported via the app vs 2 week web-based recall. Mean percent comparisons demonstrated consistent reporting of tobacco use, while Alcohol there were significant differences in mean percent of days for alcohol and discordant pair comparisons both showing higher reports for daily compared to recall responses.*

Tobacco – Active Objective

Meredith	2014	USA	60	43.00%	42	13	NR	3 group - - Regular smokers, light smokers, non-smokers	NR	Prototype smartphone attachment containing a CO sensor.	iOS	Breath CO Smokerlyzer®	*Reliability:* Expired CO measures from first and second breath using their prototype smartphone CO monitor were similar and not significantly different from each other and strongly correlated with each other (r=0.98). *Construct validity:* Expired CO measures taken with the prototype smartphone CO monitor were strongly correlated with the measures taken with the commercially available CO monitor (Smokerlyzer®)(r = .96). Expired CO measures from their prototype were found to be significantly lower than measurements from the commercially available CO monitor. A mixed factorial ANOVA revealed with the prototype smartphone CO monitor, regular smokers provided significantly higher expired CO

												measures than light smokers and both regular smokers and light smokers provided significantly higher expired CO measures than non-smokers. *Criterion validity*: Significantly higher area under the ROC curve were observed for the prototype smartphone CO monitor (94.7%, SE = 1.9%) compared to the Smokerlyzer (91%, SE = 2.5%), indicating their prototype was better able to identify recent smoking and abstinence.
Herbec	2020	UK	18	44.00%	31.9	13.4	NR	Daily smokers over 18 years old, with access to a smartphone	Instant Heart Rate' or 'Cardio'	The two highest rated, freely and publicly available heart rate apps on the major app stores. Apps measure users' heart rate by using the in-built camera in smartphones to detect changes in blood volume below the skin's surface.	NR	Participants assessed on three separate days when they performed different smoking behaviours: smoking as usual, no smoking and nicotine replacement therapy (NRT) product use, no smoking with NRT product use.	*Construct validity:* A decrease in post-noon heart rate was observed in all participants from smoking as usual days to days participants did not smoke and did not use a nicotine replacement product. A decrease in post-noon heart rate was also observed in 15 of 18 participants from smoking as usual days to days participants did not smoke but did use a nicotine replacement product. *Responsiveness:* Compared with the smoking as usual condition, mean heart rate was significantly lower in the smoking without nicotine replacement condition and significantly lower in the smoking with nicotine replacement condition.
Wong	2019	Malaysia	146	7.50%	47.9	0.85	NR	Smokers attending methadone clinics in Kuala Lumpar, aged over 18 years	Smokerlyzer & iCOSmokerlyzer	Smartphone attachment containing a CO sensor and accompanying app	NR	Expired CO measurements via the PiCO+ Smokerlyzer, Malay version of the Fagerstrom Test of Nicotine Dependence (FTND-M)	*Reliability:* Expired CO measures from first and second breaths using the CO monitor were significantly correlated with each other (r=0.94). *Construct Validity:* Mean expired CO levels were positively, but weakly correlated with nicotine dependence (r=0.22, p<0.01). *Criterion validity:* Mean expired CO levels were strongly correlated between both CO monitors (r=0.86)

McClure	2018	USA	16	75.00%	22.3	2	NR	Young daily smokers aged 15-25	My Mobile Monitor (M3)	Ecological momentary assessment app that facilitates time-stamped photo capture of readings from a separate CO monitor.	iOS	Photos of PiCO smokerlyzer measurements verified by the researchers	*Construct validity:* Moderate correlations between methods were found (r=.49). Rates of agreement between methods were consistent across study days, but missing data increased throughout the study. Most disagreement between methods occurred on the first day of the quit attempt.

Tobacco – Passive Objective

Shoaib	2016	The Netherlands	11	18.18%	NR	NR	20-45	NR	NR	Accelerometer and gyroscope data collected from smartwatches and smartphones.	Android	Self-reported smoking episodes.	*Construct validity:* Combining sensor data from multiple smartphone and smartwatch sensors and employing a two-layer hierarchical smoking detection algorithm authors able to correctly classify smoking episodes with high precision and recall (F-measure = 90-97% in person-dependent evaluations, F-measure = 83-94% for in person-independent evaluations). Their algorithm was found to correct up to 50% of misclassified smoking segments.
Qin	2017	Canada	3		NR	NR	NR	NR	NR	GPS, Wi-Fi and accelerometer data collected from smartphones	NR	Self-reported smoking behaviours	*Construct validity:* Multivariate Hidden Markov Models (HMMs) found to accurately classify and detect smoking activity when compared to self-report, with accuracy over 0.9, and an area under ROC curve (AUC) above 0.8. The results of HMMS with a single feature are less favourable than those for multivariate HMMs.
Dar	2018	Israel	40	22.50%	27.725	6.47	18-45	Recruited via Facebook	SmokeBeat	App collects sensor data from standard wearables (smartbands and smartwatches) to detect hand-to-mouth gestures, infer smoking episodes and notify users of detection in real time.	Android	Self-reported smoking episodes. Participants prompted to confirm within the app if a smoking episode had occurred.	*Construct validity:* 82.29% of smoking episodes (regardless of participant's position) were identified by their app. The system incorrectly detected and reported a smoking episode for 2.85% of the total episodes reported by the system.
